# Exciton Transfer Between Extended Electronic States in Conjugated Inter-Polyelectrolyte Complexes

**DOI:** 10.1021/acsami.3c14657

**Published:** 2024-01-30

**Authors:** Rachael Richards, Yuqi Song, Luke O’Connor, Xiao Wang, Eric A. Dailing, Arthur E. Bragg, Alexander L. Ayzner

**Affiliations:** ‡ Department of Chemistry and Biochemistry, University of California Santa Cruz, Santa Cruz, California 95064, United States; § Department of Chemistry, Johns Hopkins University, Baltimore, Maryland 21218, United States; ⊥ The Molecular Foundry, Lawrence Berkeley National Laboratory, Berkeley, California 94720,United States

**Keywords:** exciton, energy transfer, self-assembly, conjugated polyelectrolyte, polyelectrolyte complex

## Abstract

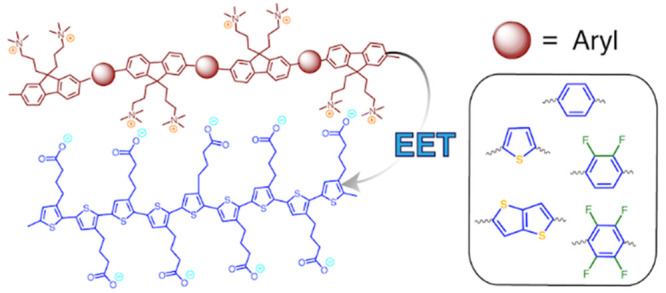

Artificial light harvesting, a process that involves converting sunlight into chemical potential energy, is considered to be a promising part of the overall solution to address urgent global energy challenges. Conjugated polyelectrolyte complexes (CPECs) are particularly attractive for this purpose due to their extended electronic states, tunable assembly thermodynamics, and sensitivity to their local environment. Importantly, ionically assembled complexes of conjugated polyelectrolytes can act as efficient donor–acceptor pairs for electronic energy transfer (EET). However, to be of use in material applications, we must understand how modifying the chemical structure of the CPE backbone alters the EET rate beyond spectral overlap considerations. In this report we investigate the dependence of the EET efficiency and rate on the electronic structure and excitonic wave function of the CPE backbone. To do so, we synthesized a series of alternating copolymers where the electronic states are systematically altered by introducing comonomers with electron withdrawing and electron-rich character while keeping the linear ionic charge density nearly fixed. We find evidence that the excitonic coupling may be significantly affected by the exciton delocalization radius, in accordance with analytical models based on the line-dipole approximation and quantum chemistry calculations. Our results imply that care should be taken when selecting CPE components for optimal CPEC EET. These results have implications for using CPECs as key components in water-based light-harvesting materials, either as standalone assemblies or as adsorbates on nanoparticles and thin films.

## I Introduction

Light-harvesting materials based on polymeric semiconductors have been the subject of research for multiple decades. The overwhelming focus has been on solution-processed organic photovoltaics cast from volatile organic solvents and deposited as thin films.^1−3^ Yet there is a complementary need to form self-assembled systems capable of eventually converting photon energy into chemical potential energy,^4−8^ and it would be desirable to do so using environmentally benign aqueous processing. Conjugated polyelectrolytes (CPEs) are attractive for such an application because, in addition to their highly delocalized electronic states, they can be made to be water-soluble and to have the potential for hierarchical self-assembly given the diversity of their noncovalent interactions.^9−16^

We previously showed that oppositely charged inter-CPE complexes (CPECs) can be electrostatically assembled in water over a broad range of ionic strengths.^17,18^ If the two CPEs comprising the CPEC are judiciously chosen to act as an exciton donor–acceptor pair, they may undergo extremely rapid electronic energy transfer (EET) from the donor to the acceptor. The EET time scale can be commensurate with natural photosynthetic pigments.^18^ Since EET is critical to all high-performance light-harvesting systems,^19−21^ CPECs can be attractive artificial light-harvesting antennae in overarching, aqueous light-harvesting systems.

Although we previously demonstrated that EET between a model donor and acceptor CPE within a CPEC could be ultrafast,^18^ it remains unclear how the EET time scale responds to changes in the chemical structures of the complexed CPEs. In particular, depending on the desired material, it may be necessary to tune the electronic bandgap or the emission spectrum to harvest a particular wavelength range. Doing so requires modification of the backbone chemical structure, which may significantly affect the CPEC excitonic coupling. That is, tuning the backbone chemistry inevitably modifies the excitonic wave function and thus the excitonic coupling that determines the EET rate.

To evaluate how the EET rate responds to changes in chemical structure, one often uses the celebrated Förster model.^22,23^ Within this model, changes in the chemical structure are encoded in the emission spectrum of the isolated exciton donor and the absorption spectrum of the isolated exciton acceptor. The key assumption implicit to this model is that the separation between the donor and the acceptor is significantly larger than the spatial extent of their excitonic wave functions. But within a CPEC, it is often the case that the separation between donor and acceptor chains is comparable to or smaller than the exciton delocalization radius, or mean chromophore length.^24,25^ In such a case, the Förster model may break down, making it challenging to determine how changes in the backbone chemical structure will influence the EET rate. This knowledge gap limits the application of these materials as energy transfer antennae.

In this article, we interrogate the dependence of EET within a CPEC on the chemical structure of the CPEs. To do so, we fixed the anionic acceptor CPE and synthesized a series of cationic, alternating copolymer donor CPEs with identical ionic monomers but differing in subtle modifications of the second comonomer. Variation of the second monomer while keeping the linear charge density nearly fixed allows us to manipulate the excitonic wave function on the donor without significantly changing the thermodynamics of electrostatic self-assembly. We then used a combination of steady-state and ultrafast optical probes to characterize the relative EET efficiencies and rates. We find that relatively subtle changes in the chemical structure of one monomer can lead to large differences in EET. We argue that the primary influence of the second nonionic monomer on the donor CPE is to alter the exciton delocalization radius, in turn, influencing the exciton transfer integral. This work provides a clear demonstration that manipulating EET between proximal conjugated polymer chains requires considerations that go well beyond the spectral overlap. Our results have direct implications for the choice of CPEC constituents in water-based light-harvesting materials.

## 2 Experimental Section

### Polymer Synthesis

**Scheme 1:**
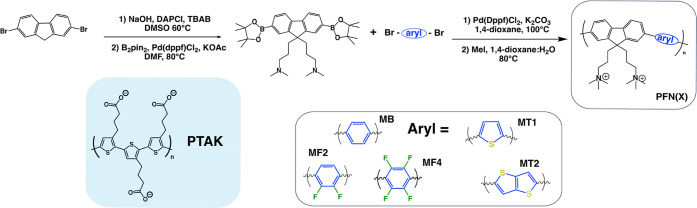
Synthetic Scheme for the PFNX Polymer Series Once synthesized, monomer FNB is then coupled to one of the aryl co-monomers shown via Suzuki cross-coupling to produce the neutral precursor polymers. The final CPE series is obtained by reacting all neutral polymers with methyl iodide.

The full synthetic details and characterization are provided in the Supporting Information. Briefly, a polyfluorene-based exciton–donor CPE series containing identical side chains but differing in their backbone chemical structures was synthesized, as shown in Scheme 1. This PFNX (X = F4, F2, B, T1, T2) series was formed via Suzuki polycondensation reactions between propyl dimethylammine-functionalized fluorene pinacol boryl ester (FNB) and the aryl comonomers, the details of which can be found in the Supporting Information. Subsequently, the resulting neutral CPEs’ pendant alkyl amines were quaternized and immediately dialyzed in water using a membrane with a 10 000 MW cutoff. Purity of all synthesized products were ensured via proton (^1^H) NMR spectroscopy, the details of which can be found in Section S1 of the Supporting Information.

### Molecular Weight Determination

To measure the molecular weights of CPEs, it is common to form neutral precursor conjugated polymers, which are eventually quaternized to form CPEs. In favorable cases, standard size-exclusion chromatography (SEC) measurements may then be doable on the neutral precursors. We were able to perform SEC measurements on nPFNF2 with tertiary dimethylamine side chains, the neutral precursor to the PFNF2 CPE. The calculated weight-average molecular weight is ∼140 000 g/mol, and the polydispersity is ∼1.7. Such a MW corresponds to a degree of polymerization of ∼311. The molecular weight distribution for nPFNF2 is shown in the Supporting Information in Figure S7. Unfortunately, the other neutral precursor polymers were insufficiently soluble in both warm THF and hot (150 °C) trichlorobenzene to obtain data of comparable quality to nPFNF2. However, we note that all of the synthetic procedures for all donor CPEs were identical. Thus, we believe it is reasonable to expect that the molecular weights of all of the donor polymers are comparable.

### Sample Preparation

The anionic CPE poly(butylcarboxythiophene) (PTAK) with a MW of 16 000 Da was obtained from Rieke Metals and used as received. Stock solutions of PFNX CPEs and PTAK (3.0 mg/mL) were prepared in HPLC-grade water (Sigma-Aldrich) and complexed in the desired molar ratios to form CPECs. To ensure optical clarity, the PTAK stock solution was stirred at ∼70 °C for 24 h and the PFNX stock solutions were stirred at ∼70 °C for 72 h. Exposure to ambient light was minimized, and CPEC solutions were vigorously degassed with argon prior to optical measurements. CPEC solutions with PFNX/PTAK at a charge ratio 70:30 (PFNX:PTAK) were prepared based on the number of charges per monomer unit, as the PFNX monomer carries a charge of 2+, and the ionized PTAK monomer carries a 1– charge. Specifically, 0.25 mg/mL PFNX and 0.1072 mg/mL PTAK solutions were mixed to make samples. PFNX was added directly to PTAK at the desired charge ratio. CPEC solutions were then stirred at 70 °C for 24 h.

### Steady-State Spectroscopic Measurements

Optical density (absorption) measurements were collected from the above solutions using a Shimadzu UV-2700 spectrometer. Spectra were collected over the 300–800 nm wavelength range in 1.0-nm increments, with a 1 mm path length quartz cuvette. Photoluminescence and photoluminescence excitation spectra were collected by using a Horiba Fluoromax-4 spectrometer in a right-angle geometry. In photoluminescence measurements, the excitation wavelength was set to each respective CPE’s peak absorption wavelength, ranging from 330 to 445 nm for PFNX donors and 550 nm for the acceptor PTAK. The PL intensity was then collected in the 350–800 nm range in 1-nm increments, with excitation and emission slit widths set to 1 nm bandpass. For photoluminescence excitation measurements, the fixed emission wavelength was set to 715 nm, and the excitation wavelength was scanned from 300 to 800 nm in 1-nm increments, with excitation and emission slit widths set to 2 nm bandpass.

### Time-Resolved Photoluminescence

Time-correlated single photon counting (TCSPC) measurements were carried out on a home-built setup, which has been described in detail in previous work.^9^ Briefly, the excitation source was a pulsed picosecond SuperK EXTREME (NKT Photonics) supercontinuum laser coupled to a SuperK SELECT (NKT Photonics) acousto-optic filter and an external RF driver (NKT Photonics) to select the wavelength of the excitation pulse. With the supercontinuum laser, both the native thiophene-containing subfamily and the corresponding CPEC samples were excited at wavelengths relative to the donor and acceptor PTAK: 425 and 600 nm, respectively. Some PFNX samples were excited at 375 nm by a pulsed picosecond diode laser (BDS-SM Series, Becker and Hickl, GmbH). Emission was measured on a hybrid photomultiplier tube (Becker and Hickl, GmbH). The signals were then sent to a Simple Tau SPC-130 (Becker and Hickl, GmbH) for initial data visualization and analysis. Long-pass filters were used on the detection arm with a 400, 475, or 590 nm onset depending on either the donor or acceptor excitation wavelength in CPEC or native CPE solutions. Subsequently, the monochromator was set to collect emission intensity at 410, 475, and 620 nm. All measurements were taken with a right angle Starna Cell quartz cuvette, while the excitation and detection Glan-Thompson polarizers were offset from each other by the magic-angle (54.7°) to minimize polarization effects.

### Small-Angle X-ray Scattering

SAXS measurements were conducted at the Stanford Synchrotron Radiation Lightsource at beamline 4–2. This beamline is equipped with a Pilatus 3X detector and a robotic autosampler that feeds samples from a 96-well plate to a thin-walled quartz capillary cell. To avoid degradation, each sample was oscillated for the duration of exposure to X-ray radiation. The samples were irradiated with 10 consecutive 1-s exposures at 11 keV at a sample-detector distance of 1.7 m. This setup yielded an effective *Q*-range of 0.0068–0.67 Å^–1^. With the aid of the SAStools software suite, averaged, totaled, and background subtracted data sets were used in analysis and plotting of SAXS data.

### Ultrafast Transient Absorption Spectroscopy

A general description of our setup for transient absorption measurements has been provided in detail elsewhere.^26^ Here we discuss specific experimental details of the work presented. Ultrafast excitation and probe pulses were generated using the amplified output of a Ti: sapphire laser (Coherent Legend Elite, 800 nm center wavelength, ∼35 fs pulse duration, 3.5 mJ/pulse, 1 kHz repetition rate). Excitation pulses at 400 nm were generated by second harmonic generation (SHG) of the 800 nm laser output in a BBO crystal. Excitation pulses at 360 and 600 nm were generated through fourth harmonic and second harmonic generation with the NIR signal from an optical parametric amplifier (OPA, Coherent OperaSolo). Broadband (continuum) probe pulses (450–750 nm) were obtained via white-light generation in a 2 mm sapphire crystal driven by a few nanojoules of the 800 nm laser fundamental. Probe pulses were transmitted through a wire-grid polarizer (Thorlabs) set at the magic angle (54.7°) with respect to pump-pulse polarization. The polarizer is the last probe optic before the sample and is used to eliminate time-dependent polarization effects in transient absorption spectra. The path of the pump beam was aligned via a corner-cube retroreflector mounted to the carriage of a motorized translation stage (Newport); a time delay between pump and probe pulses ranging from −10 to 1400 ps was obtained by varying the carriage position. The effective time resolution of our experiments was determined to be 160 fs based on the resolution limited rise of long-lived donor CPE excited-state features (*vide infra*). We used excitation fluences that ensured limited fluence dependence in measured signals; for 360 and 400 nm, we found weak fluence dependence below 4 mJ/cm^2^, whereas fluences below 20 uJ/cm^2^ were used at 600 nm excitation due to a greater sensitivity to fluence at this excitation wavelength.

### Computational Details

To calculate the center-to-center distance between the donor and acceptor backbones, the PFNX:PTAK complexes were fully relaxed with the semiempirical quantum mechanical method GFN2-xTB with balanced treatment of noncovalent interactions, including multipole electrostatics and density-dependent dispersion contributions.^27^ Each complex was modeled with a single PFNX donor chain with four repeat units and a single PTAK acceptor chain with five repeat units. The calculations were performed using the DFTB+ software package^28^ and the relaxed structures were visualized using the VESTA program.^29^ To calculate refined structures and transition properties of PFNX donors, density functional theory (DFT) calculations were performed using the ORCA quantum chemistry package.^30^ The ground-state structures of isolated repeat units for PFNX were fully optimized with the range-separated hybrid functional ωB97X-D3 that incorporates the DFT-D3 dispersion correction and the diffuse augmented def2-TZVPD basis set.^31,32^ Time-dependent DFT (TDDFT) calculations with the same density functional and basis set were performed on the optimized geometries to determine the oscillator strengths and transition dipole moments of their lowest excited states. Transition densities and charge difference densities were computed to visualize the characteristics of the electronic transitions. The visualization plots of charge differences, transition densities, and natural transition orbitals were produced using the VMD program.^33^

## 3 Results

In this paper, we focus on oppositely charged CPECs composed of variable cationic CPEs, which act as exciton donors, and a common anionic exciton-acceptor CPE. The synthesized exciton-donor CPE set is composed of a series of alternating copolymers, abbreviated PFNX, containing chemically identical charged fluorene monomers but differing in the chemical structure of the comonomer. Here X refers to the variable monomer in the alternating copolymer series. The chemical structures of all CPEs are shown in Scheme 1. The specific variable monomer choice was motivated by the following. (1) We desired to systematically vary the electronic wave function along the donor backbone in a tractable manner while ensuring that there would be spectral overlap with the fixed exciton acceptor. (2) We aimed to keep the donor counterion identity and linear ionic charge density along the donor contour very similar across the series. Doing so helps ensure that the electrostatic free energy of interpolyelectrolyte complexation, which includes both the interpolyelectrolyte binding energy and the change in entropy upon counterion release, are similar across the series. The linear charge densities of the donor CPEs are (in units of e/Å) 0.34, 0.34, 0.34, 0.36, and 0.28 for PFNF4, PFNF2, PFNB, PFNT1, and PFNT2, respectively.

The common acceptor CPE, PTAK, is a regioregular polythiophene derivative with butylcarboxylate side chains. The choice of PTAK as the anionic acceptor CPE was partially motivated by the fact that its homopolythiophene backbone is relatively simple compared to other reasonable anionic CPEs that could function as exciton acceptors. Additionally, the PTAK PL quantum yield (PLQY), while low in isolation due to its collapsed coil structure, becomes substantially higher upon complexation to the donor due to the extension of the PTAK backbone.^18,34^

We believe that at relatively early times after being formed (of order a few days), the complexes are to a good approximation largely composed of at most a few oppositely charged chains. This is consistent with the lack of significant light scattering from CPEC solutions shortly after complexation. Scattering from fresh CPEC solutions was evaluated by measuring the sample turbidity. Here turbidity is defined as the negative logarithm of the ratio of the transmitted to the incident light intensity at a nonabsorptive wavelength, which was 800 nm in this work. We find that the turbidity in fresh CPEC solutions is negligible; the scattered light intensity is expected to increase monotonically with decreasing wavelength, and the CPEC absorption spectrum is effectively seen to decay to zero by 650 nm (*vide infra*). Furthermore, we expect the thermodynamic driving force for CPEC formation across the CPE series to be dominated by electrostatic side chain interactions and the increase in solution entropy due to counterion release upon complexation. Because of these factors, we believe that the average number of chains per complex is similar and likely near two. Thus, care was taken to ensure that all measurements were performed relatively shortly after preparation of CPEC solutions.

Below we first interrogated the relative EET efficiencies by analyzing the photoluminescence (PL) excitation spectra of the complexes. We then measured the ultrafast EET dynamics using pump/probe spectroscopy. Finally, we characterized potential differences in the CPEC structure using both structural and optical probes.

### 3.1 Steady-State Photophysics

**Figure 1:**
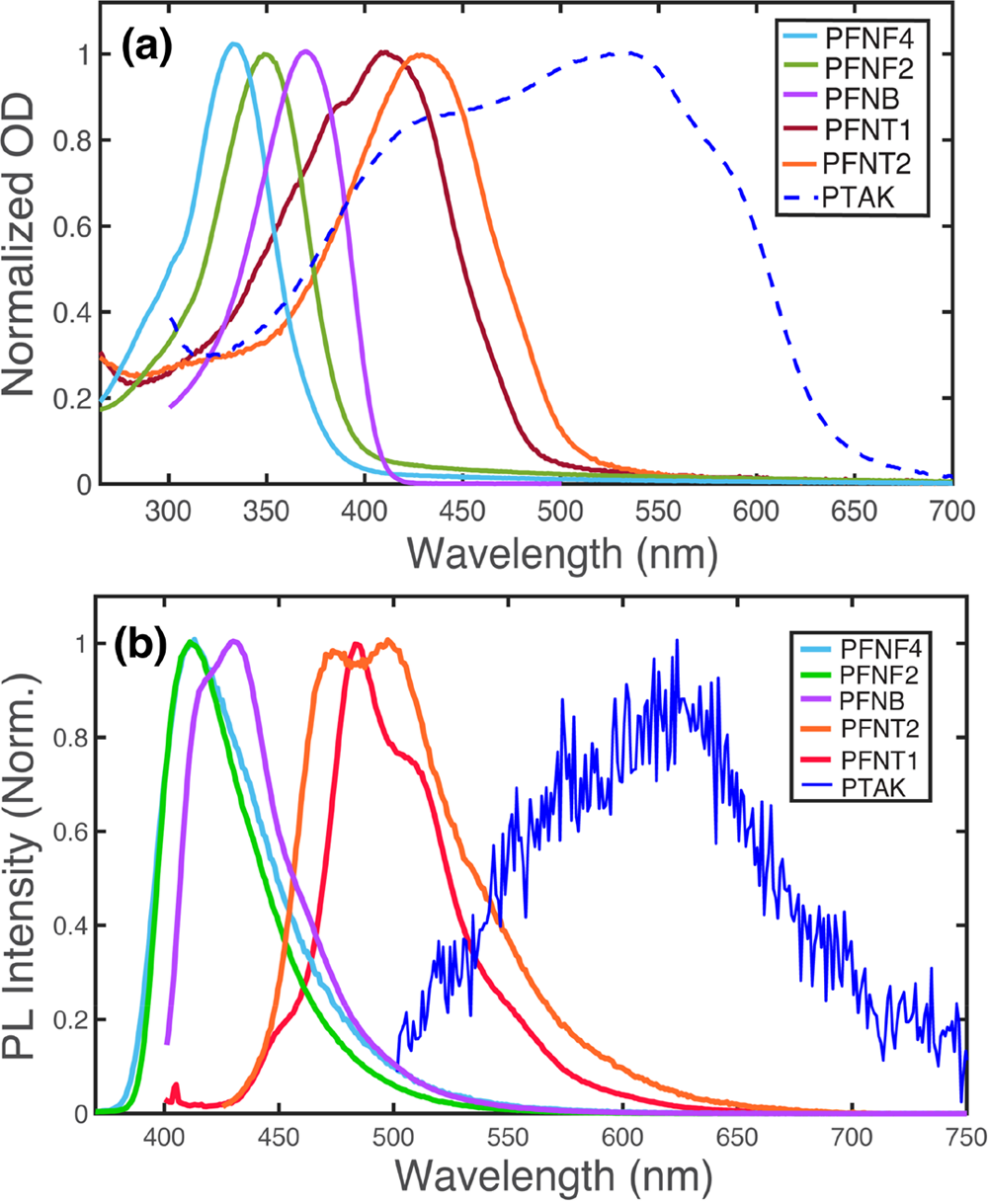
Optical properties of isolated donor and acceptor CPEs. (a) Normalized absorption (OD) spectra. (b) Normalized PL spectra. The progression in peak OD wavelength illustrates the systematic variation in the bandgap and thus the electronic states comprising the low-lying exciton transition.

Figure 1a shows the peak-normalized ground-state absorption spectra (or optical density, OD) of both the native anionic acceptor CPE, PTAK, and the isolated cationic donor CPE series, PFNX, in dilute, salt-free aqueous solution. By keeping the ionic fluorene monomer fixed and varying the other monomer, we aimed to systematically alter the electronic states and thus the bandgap along the polymer series without inducing significant changes in the ionic linear charge density of the CPEs. The progression in the band gap is clearly demonstrated in the shift in λ_max_ among the donor CPEs. It is convenient to partition the PFNX donor series into two subfamilies: (i) the poly(fluorene-*alt*-phenylene) family, with increasing fluorine content on the phenyl monomer (PFNB, PFNF2, PFNF4), and (ii) the thiophene family containing poly(fluorene-*alt*-thiophene) (PFNT1) and poly(fluorene-*alt*-thienothiophene) (PFNT2). In what follows, we will refer to the polymers by their shorthand X monomer abbreviation.

The phenyl subfamily CPEs exhibit absorption spectra that are quite similar, only differing in a slight blueshift of approximately 20 nm between the polymers: λ_max_ for B, F2, and F4 was 375, 348, and 332 nm, respectively. This corresponds to a slight increase in the band gap as a function of increasing fluorine substitution. The λ_max_ for the two CPEs in the thiophene subfamily are shifted by ∼30 nm: 405 and 435 nm for T1 and T2, respectively. The change in bandgap reflects both a change in the electronic structure of the variable monomer and differences in the torsional potential. The latter can influence the delocalization radius of the exciton and thus the splitting between frontier orbital energy levels.^35^

Figure 1b shows normalized PL spectra of the exciton donor series and the common exciton acceptor in isolated aqueous solution. Interestingly, although the OD peaks for fluorinated polymers are blueshifted with respect to B, the F2 and F4 PL peaks are effectively on top of each other. The T1 and T2 emission bands also encompass a comparable wavelength range albeit with substantially different apparent vibronic structure. All donor emission bands have spectral overlap with the PTAK absorption spectrum, which we quantify below. The native PTAK PL spectrum lies to the red of the donor polymers as expected. The large noise level given the comparable solution concentration reflects the fact that the native conformation of regioregular PTAK chains is highly coiled, leading to a very low PLQY.^34^

**Figure 2:**
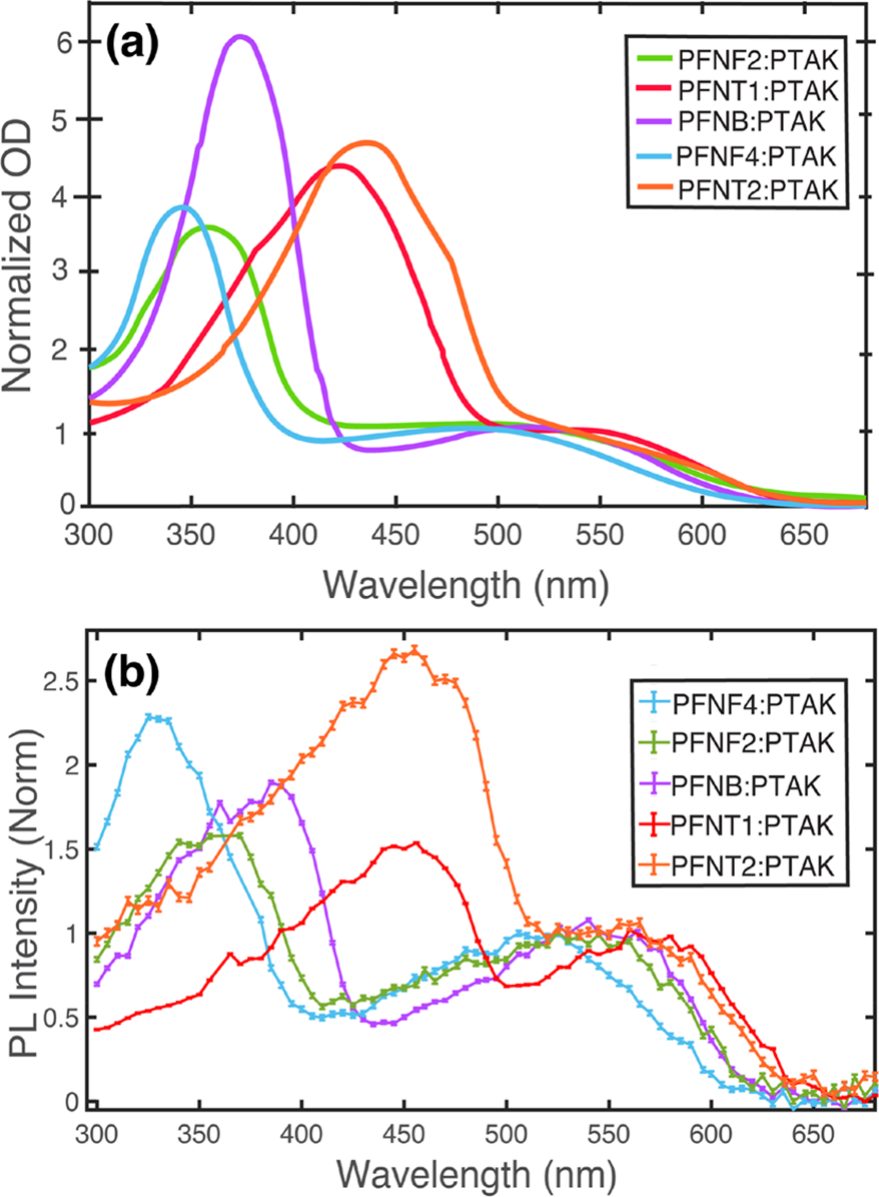
Optical spectroscopy of CPECs. (a) OD spectra normalized to the PTAK absorption band on the red side. (b) PL excitation (PLE) spectra collected at a fixed emission wavelength on the red side of the PTAK PL spectrum. Spectra have been normalized on the red side, exclusively corresponding to direct PTAK excitation. The specific choice of the fixed wavelength was made to ensure a vanishing probability that donor PL would be detected. The shape of the PLE spectra provide unambiguous evidence of EET. The contribution on the blue side due to EET from the donor can be isolated and used to quantify a relative EET efficiency.

Having characterized the OD and PL spectra of isolated CPE solutions, we went on to form CPECs. This was done by combining the polymers at the fixed molar polycation: polyanion charge ratio of 70:30, respectively, as described in more detail in the Experimental section. Thus, in all cases, the donor polymer was in molar excess. This choice was made for the following reasons: (i) In our previous work, we showed that this CPEC ratio produced extended PTAK chains within the complex with a PLQY that increased substantially compared to the isolated PTAK solution. (ii) Having the acceptor CPE be the limiting component minimizes the probability that any PTAK chains remain uncomplexed. This in turn allowed us to use PL excitation spectroscopy to compare relative EET efficiencies (*vide infra*) by monitoring PTAK PL signal. Figure 2a shows OD spectra of CPECs prepared with all the donor CPEs and the common acceptor CPE. The spectra show the characteristic peak for each of the donor and acceptor components of the CPEC. The absorption bands spanning the 300–550 nm range correspond to the PFNX donors, while the broader peak centered about 525 nm corresponds to PTAK.

We then proceeded to characterize the steady-state EET efficiency, *E*, within the CPEC across the donor CPE series. The common way of calculating *E* from steady-state measurements is to calculate the ratio of the difference in PL intensity of the donor when it is isolated vs in the presence of acceptor relative to the donor PL signal in isolation.^36^ We believe that this method may lead to inaccuracies for CPECs because it relies on assuming that the PLQY of the donor is unchanged when it is electrostatically bound to the acceptor. In the CPEC this is likely a severe assumption, as complex formation may lead to differences in the ensemble of thermally accessible conformations of the donor polymer and thus its PLQY. Our previous measurements support this concern.^18^ Instead, it is desirable to extract *E* directly from the spectra of the complex without the need to compare these to control donor-only measurements.

To bypass the need to rely on such an assumption, we turned to PL excitation (PLE) spectroscopy. In this measurement, we fixed the emission wavelength on the red side (715 nm) of the PTAK PL spectrum to ensure that this wavelength was significantly red-shifted relative to the tail of all the donor PL spectra. The acceptor PL intensity was then measured as a function of excitation wavelength *λ*_*ex*_ across the CPEC OD spectrum from 300 to 700 nm, encompassing both the donor and acceptor absorption windows. The choice to limit the lower bound to 300 nm was made largely to avoid the region where the S_0_ → S_2_ absorption band of PTAK, T1, and T2 begins to acquire substantial amplitude.

PLE spectra for all PFNX:PTAK CPECs are shown in Figure 2b. It is clear that the shape of the PLE spectrum qualitatively resembles the corresponding OD spectrum of CPEC for all donor CPEs. Since only PTAK emission is monitored in this experiment, it is expected that the PLE spectrum on the red side will reflect the OD spectrum of PTAK. The fact that the PLE spectrum clearly traces out the donor OD spectrum on the blue side is unambiguous evidence of EET from the exciton donor to the exciton acceptor. To compare *E* across the donor series, we first normalized PLE spectra to the intensity in the acceptor-only region, i.e. in the region to the red of ∼525 nm, and then attempted to isolate the donor contribution to the PLE, . This procedure provides not an absolute but a relative EET efficiency, *E*_rel_, which can be defined as
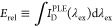
1where the limits of integration encompass the 300–700 nm range. Nevertheless, this approach would allow us to elucidate how varying monomer X while keeping the rest of the donor CPE chemical structure fixed influenced *E*.

Estimating *E*_rel_ from the PLE spectra requires some care. This is because calculating  necessitates subtracting the contribution to the PLE that comes from native PTAK PL within the complex. That is, there is nonzero PTAK absorption at excitation wavelengths where the donors primarily absorb, giving rise to a PTAK PLE background due to direct excitation of PTAK. The PLE signal due to direct PTAK excitation must then be subtracted off, but this presents a complication: On the blue side of the PLE spectrum we do not know the precise functional form of the acceptor PL signal due to direct acceptor excitation, (*λ*_*ex*_). Our approach was to estimate the wavelength dependence of  in the blue by using the Gaussian fit parameters of an isolated PTAK OD spectrum as an initial guess for a constrained fit of the red side of the PLE spectra. The PLE fitting range was bounded from below by the excitation wavelength above which the donor contribution went to zero. The detailed procedure used to estimate  is provided in Section S3 of the Supporting Information.

The PLE fits together with the isolated PTAK OD spectrum are shown in Figures S8–S17 of the Supporting Information. It is clear that the red side of the PLE spectrum fits very well for all CPECs. Subtracting the PLE fit from the measured PLE spectrum gives us the contribution to PTAK PL that comes from exciting the donor CPEs, (*λ*_ex_), *i.e*., the contribution due to EET. Despite the fact that the fits on the red side of the spectrum are of a good quality, there is intrinsically an error associated with inferring (*λ*_ex_) in the donor region, which will give rise to an error associated with estimating the donor contribution and thus an error in *E*_rel_. To get a rough estimate for this error, we subtracted the (*λ*_ex_) fit from the PTAK OD spectrum (both normalized to the PTAK peak on the red side) to form a difference curve, the magnitude of which at every wavelength we associate with the standard deviation of (*λ*_*ex*_). Section S3 of the Supporting Information describes our rationale and justification for this means of estimating the error and the background intensity on top of which (*λ*_ex_) sits. We note that setting the lower limit on the integral in (*λ*_ex_) to 300 nm will almost certainly preferentially *understimate* this quantity for F4 and F2. Nevertheless, we believe this is the appropriate conservative choice if one wants to systematically avoid having to account for potential EET between higher-energy excitonic states.

To obtain insight into the ordering of *E*_rel_ across the donor series, it is desirable to compare it to the ordering predicted by the celebrated Förster EET model. The model assumes that the average distance between the donor and the acceptor is large compared to the spatial extent of the excitonic wave functions. The excitonic coupling is then assumed to be described by a dipole–dipole interaction between the point transition dipole moments of the donor and the acceptor. A natural length scale characterizing *E* called the Förster radius *R*_*0*_ emerges; at this distance the Förster EET rate and the radiative relaxation rate are equal.^37^*R*_0_ is determined by
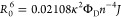
2Here, *R*_0_ is in nm, κ^2^ is the transition dipole orientation factor, Φ_D_ is the PLQY of the donor, *n* is the refractive index of the medium taken to be that of pure water, and *J* is the spectral overall integral given by
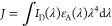
3where *I*_D_ is the PL spectrum of the isolated exciton donor normalized to unit area, *ε*_A_ is the extinction coefficient spectrum of the isolated acceptor (monomers) in units of M^–1^ cm^–1^, and λ is in nm.

The transition dipole orientation factor κ^2^ ranges from 0 to 4, where a value of 1 corresponds to parallel transition dipoles, 2/3 represents a random orientation, and 4 is a consequence of dipoles that are both parallel and collinear.^38^ In this work, we set κ^2^ to 1 for all Förster model calculations. We believe this is the least biased choice as it is expected that the donor and acceptor CPEs assemble in an approximately parallel geometry. Such local complex structures are consistent with structures obtained to calculate mean separations between donor and acceptor CPE backbones (Figure S18).

The Förster EET efficiency *E*^F^ is related to the Förster radius via
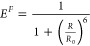
4where *R* is separation between the donor and the acceptor. To estimate *R*, we found the center-to-center distance between the backbones by using semiempirical quantum mechanical calculations. Results from these calculations are shown in Table S2 of the Supporting Information. The calculated values of *E*_F_ and the estimated values of *E*_rel_ are shown in Table 1. We see that the ordering of *E*_rel_ does not follow that of *E*^F^.

**Table 1:** Measured and Calculated Quantities Used to Compute Relative EET Efficiencies and Predictions from the Förster Model

CPE	PLQY (%)	Spectral overlap integral (*J*) (× 10^12^)	Förster radius (*R*_0_) (Å)	Donor–acceptor distance (*R*) (Å)	FRET efficiency (*E*) (%)	*E*_rel_	*E*_norm_ (× 10^–11^)
PFNF4	43	2.29	17.3	13.0	84.7	142 ± 7	6.18
PFNF2	44	2.53	17.7	12.7	87.9	99 ± 11	3.91
PFNB	41	2.93	17.9	13.2	86.1	139 ± 14	4.27
PFNT1	69	4.81	21.2	13.7	93.2	132 ± 19	2.74
PFNT2	67	5.60	21.6	13.1	94.7	218 ± 22	3.89

For incoherent exciton transfer described by the Fermi Golden Rule, the EET rate scales as the product of the square of the excitonic coupling, *V*_EX_, and a factor that ensures energy conservation between the initial and final states participating in the EET process. The latter is related to *J*, which tracks the position of the donor emission spectrum relative to that of the acceptor OD. Arguably the more interesting quantity is . Here Ψ_D_ is the wave function of the donor, Ψ_A_ is that of the acceptor, *V̂* is the operator that describes the electronic coupling between the donor and the acceptor, and * indicates an excited state. It is *V*_EX_ that will reflect the precise excitonic wave functions of the donor and the acceptor and thus will encode the dependence of the delocalized electronic states on monomer X. It is instructive to divide out *J* to form a normalized (relative) EET efficiency *E*_norm_*= E*_rel_/*J*, the quantity most directly related to (the square of) *V*_EX_. Doing so implicitly assumes that the donor exciton population undergoes rapid relaxation within its density of states before EET takes place since *J* is based on the steady-state PL spectrum of the donor. Interestingly, although *E*_rel_ is the largest for T2, we find that *E*_norm_ is the largest for F4.

### 3.2 Ultrafast Energy Transfer Dynamics

Having characterized the relative EET efficiency via steady-state measurements, we directly investigated the time scale over which EET occurred across the donor series. In our previous work, we showed that excitons were transferred from the donor PFNB to the acceptor PTAK in approximately 240 fs.^4^ Since we previously characterized EET dynamics in the PFNB:PTAK CPEC, below we focus on complexes involving the remaining members of the donor CPE set.

**Figure 3:**
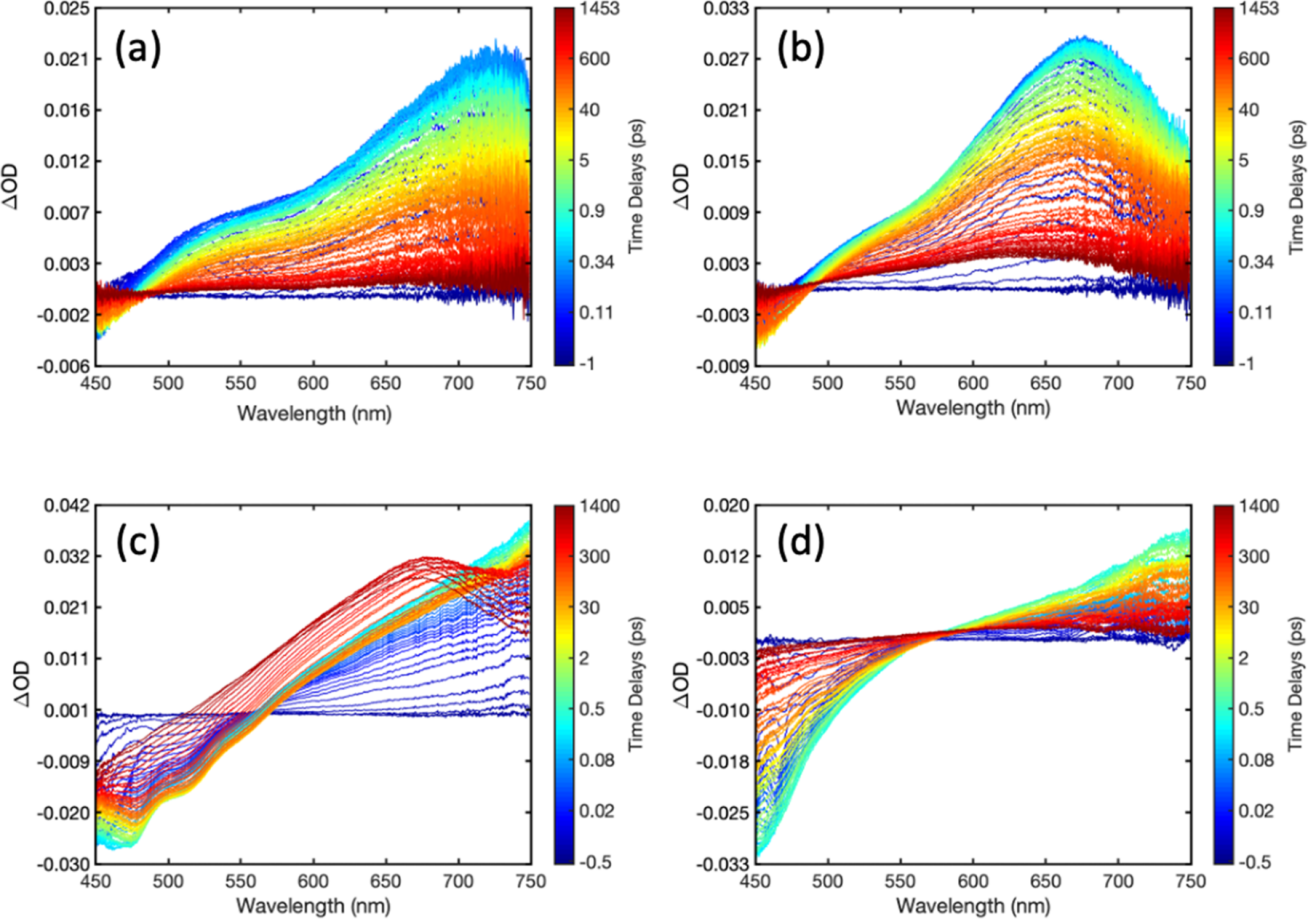
Transient absorption spectra obtained with uncomplexed (isolated) donor CPEs: (a) F4, (b) F2, (c) T1, and (d) T2. Spectra were collected following excitation near the peak of the steady-state absorption spectrum of each donor (360 nm for F4 and F2; 400 nm for T1 and T2). Spectral dynamics are explained in the text. The same data are presented as false-color contour plots in Figure S19.

Figure 3a–d presents transient absorption spectra measured at various pump–probe delays of the dilute aqueous solutions of the donor CPEs (isolated/no acceptors present). The color gradient scale to the right of each panel maps to the pump–probe time delay in ps. (A corresponding false-color contour representation of these data is presented in Figure S20.) The uncomplexed polymers were excited near their absorption maxima: 360 nm for the phenyl subfamily (F2 and F4), and 400 nm for the thiophene subfamily (T1 and T2). All pump–probe spectra are composed of primarily two features in the wavelength range probed (450–750 nm): (1) negative signal arising from stimulated emission of the excited donor, which has a spectral profile that approximately matches the steady-state PL spectrum of each donor; and (2) positive signal due to excited-state transient absorption (TA) from the low-lying exciton state S_1_ to higher-lying states S_n_. For T1 and T2, the stimulated emission signal is likely to be overlapped partially with ground-state bleach (GSB), which lies to the blue of the emission signal. For F2 and F4, only the very red tail of the stimulated emission is observed in the spectral range probed.

The time-dependent spectral evolution for the donor CPEs following photoexcitation appears to be qualitatively similar, involving a decay of excited donor absorption and stimulated emission over 10s-100s of picoseconds. However, the spectral evolution of PFNT1 appears to be qualitatively different from the rest of the polymers, with a rise in excited-state absorption signal in the region of ∼650–700 nm and a distinct blueshift of the absorption maximum with a corresponding reduction in the stimulated emission (450–650 nm). This spectral evolution is characteristic of intersystem crossing (ISC) from singlet to triplet excitons. We expect that the yield for ISC will be greater for thiophene-containing conjugated polymers compared to the phenyl subfamily because of an increased spin–orbit coupling due to the presence of relatively heavy sulfur atoms. Coupling to the triplet manifold is likely promoted by intraring nuclear motions of the thiophene monomer. Close inspection of transient spectra for the other donor CPEs 1 ns after excitation, which reveals a weak and somewhat blue-shifted absorption relative to that of the initially populated singlet excited state, suggests that ISC may take place in all donor CPEs, but with ISC quantum yields that are less significant than those of excited T1. We hypothesize that the rigidity of the fused bithiophene ring in T2 reduces vibrationally enhanced coupling, thereby lowering the ISC rate relative to T1.

**Figure 4:**
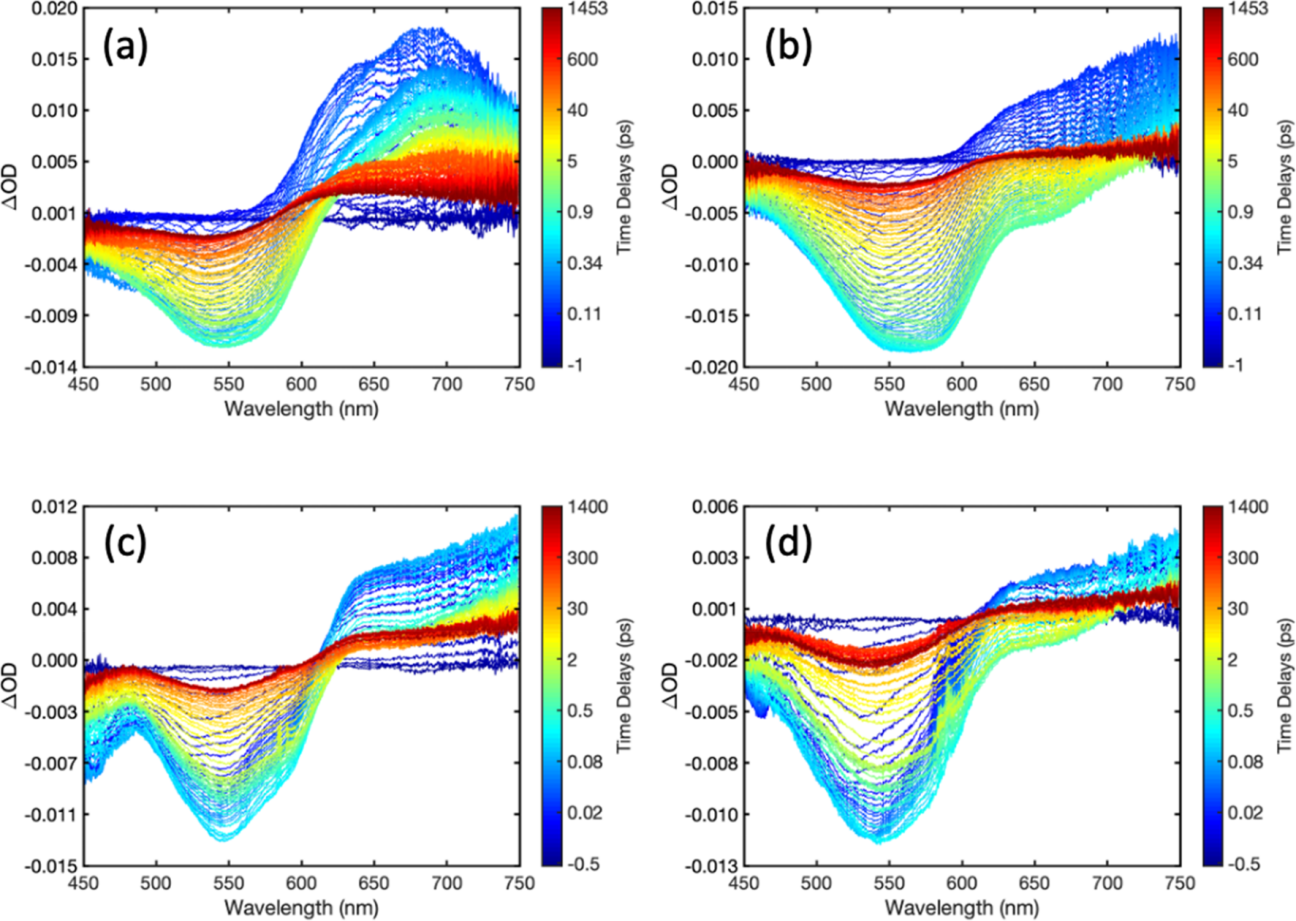
Transient absorption spectra obtained with PFNX:PTAK CPECs (70:30 ratio): (a) F4, (b) F2, (c) T1, and (d) T2. Spectra were collected following excitation near the peak of the steady-state absorption spectrum of each donor (360 nm for F4 and F2; 400 nm for T1 and T2). Spectral dynamics are explained in the text. The same data are presented as false-color contour plots in Figure S21.

Figures 4a–d presents pump–probe spectra obtained with the PFNX:PTAK CPECs, which were collected under conditions identical to those used with solutions of uncomplexed donor CPE. (A false-color contour representation of these data is presented in Figures S20 and S21.) In addition to the donor features seen in Figures 3a–d, there are additional contributions from PTAK GSB and stimulated emission spanning from below 500 nm to above 650 nm that appear rapidly after photoexcitation (*vide infra*).^9^ We cannot discount the possibility that the appearance of PTAK features in transient spectra may arise from both EET from the donor CPE and direct excitation of PTAK chromophores within complexes, given that the ground-state absorption of the uncomplexed acceptor extends down to 360 nm. However, the donor absorption dominates the absorption spectrum of CPECs with F2/F4 and T1/T2 at 360 or 400 nm, respectively, such that we expect that the rapid appearance of these significant features arises predominantly from EET.

**Figure 5:**
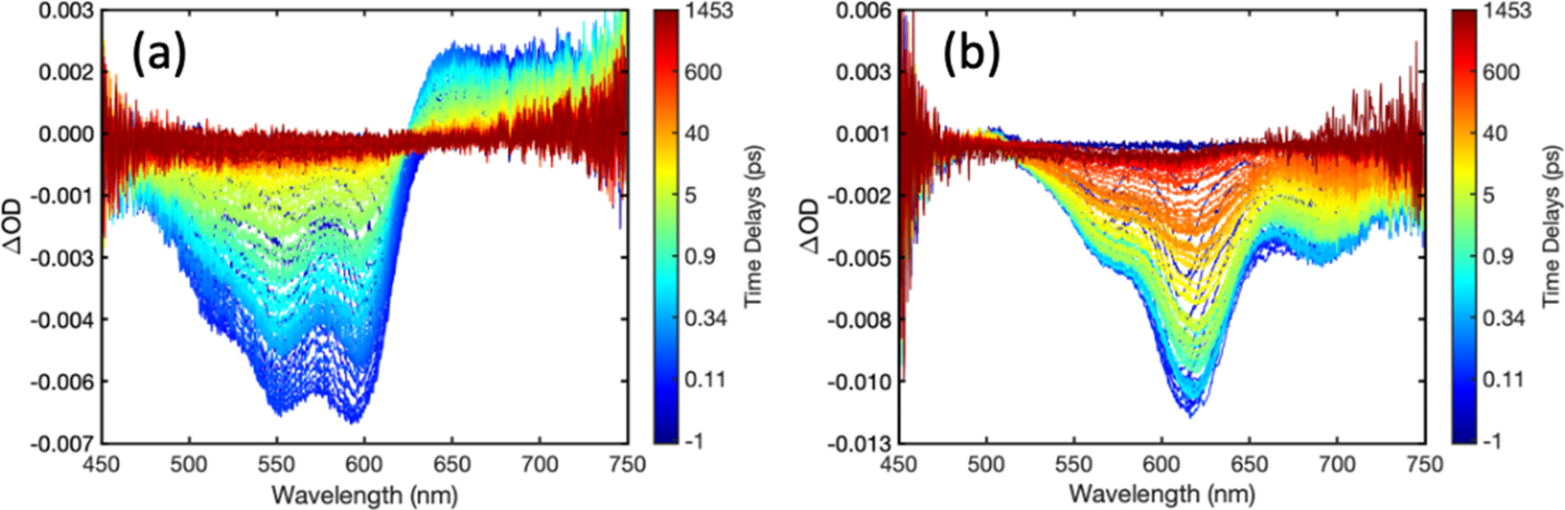
Transient absorption spectra obtained for (a) isolated PTAK and (b) a representative CPEC (PFNT1:PTAK) excited at 600 nm, which exclusively excited PTAK. Spectral dynamics are explained in the text. Data for all CPECs excited at 600 nm are presented in Figure S22.

To assess whether the observed spectral dynamics reflect EET, we examined the signal time dependence at wavelengths that correspond to TA features associated with the donor CPEs. When compared to the TA spectra of uncomplexed donors, we find a pronounced, rapid decrease in transient signals attributable to the excited donor in multiple spectral regions immediately following excitation. The clearest differences are observed in the blue at ∼450 nm, a region that contains contributions from stimulated emission from donor CPEs, as well as in the 600–750 nm range, the region of donor TA (Figure 3) and where signal due to directly excited PTAK is minimal (c.f. Figure 5 below).^9^ Most notably, for T1 and T2 CPECs, there are rises at 450 nm which correspond to a rapid (subpicosecond) reduction in the donor stimulated emission. The spectral dynamics that occur at 650–700 nm likewise reflect a subpicosecond drop in the donor TA. The dynamics in this spectral region are particularly notable for F2 and T2: For these complexes, we observe an ultrafast flip from positive to negative signals, consistent with an interconversion from excited donor CPEs (absorption) to excited PTAK (stimulated emission). Given that all four complexes were prepared at exactly the same donor:acceptor ratios, the fact that such an ultrafast flip from positive to negative signal is not observed for F4 and T1 complexes likely indicates that there are chain regions for these donors that are not fully complexed to the acceptor. Charged donor side chains in such regions likely have partially condensed counterions as described by the Manning-Oosawa model.^39^ The acceptor’s stimulated emission subsequently disappears as a result of PTAK exciton dynamics; we speculate that there may be some PTAK-to-PFNX electron transfer, similar to what has been observed for PFNB previously, based on the relative energies of donor and acceptor frontier orbitals.^40,41^

In order to examine spectral contributions from directly excited PTAK, we also studied the photophysics of CPECs excited directly at 600 nm, which selectively excites the PTAK component, and compared these to the photophysics of uncomplexed PTAK. Transient absorption data for PTAK and the PFNT1:PTAK complex are presented in Figure 5. (TA data for all complexes excited at 600 nm are presented in Figure S22.) As described previously, the spectrum of uncomplexed PTAK exhibits signatures of both H-like excitons and “free coil” states; excitation at 600 nm is selective for the former and gives rise to the characteristic polaron-pair absorption feature at 650 nm that is associated with charge separation in π-stacked polymer regions.^18^ In contrast, polaron-pair absorption is not observed for PTAK in a CPEC when excited at 600 nm; rather, the region of 650–700 nm is dominated by stimulated emission. This difference and the change in the vibronic structure of the PTAK bleach with complexation reflect an isolation of PTAK chain segments, as discussed in more detail below. The stimulated emission observed with 600-nm excitation matches the feature observed within a few hundred femtoseconds following donor excitation (F2 and T2). Notably, the polaron-pair absorption feature observed for uncomplexed PTAK (650–700 nm) has a much slower spectral evolution (decay) than the ultrafast spectral evolution observed in this region for all CPECs, supporting the assignment of the latter to PFNX-to-PTAK EET.

**Figure 6:**
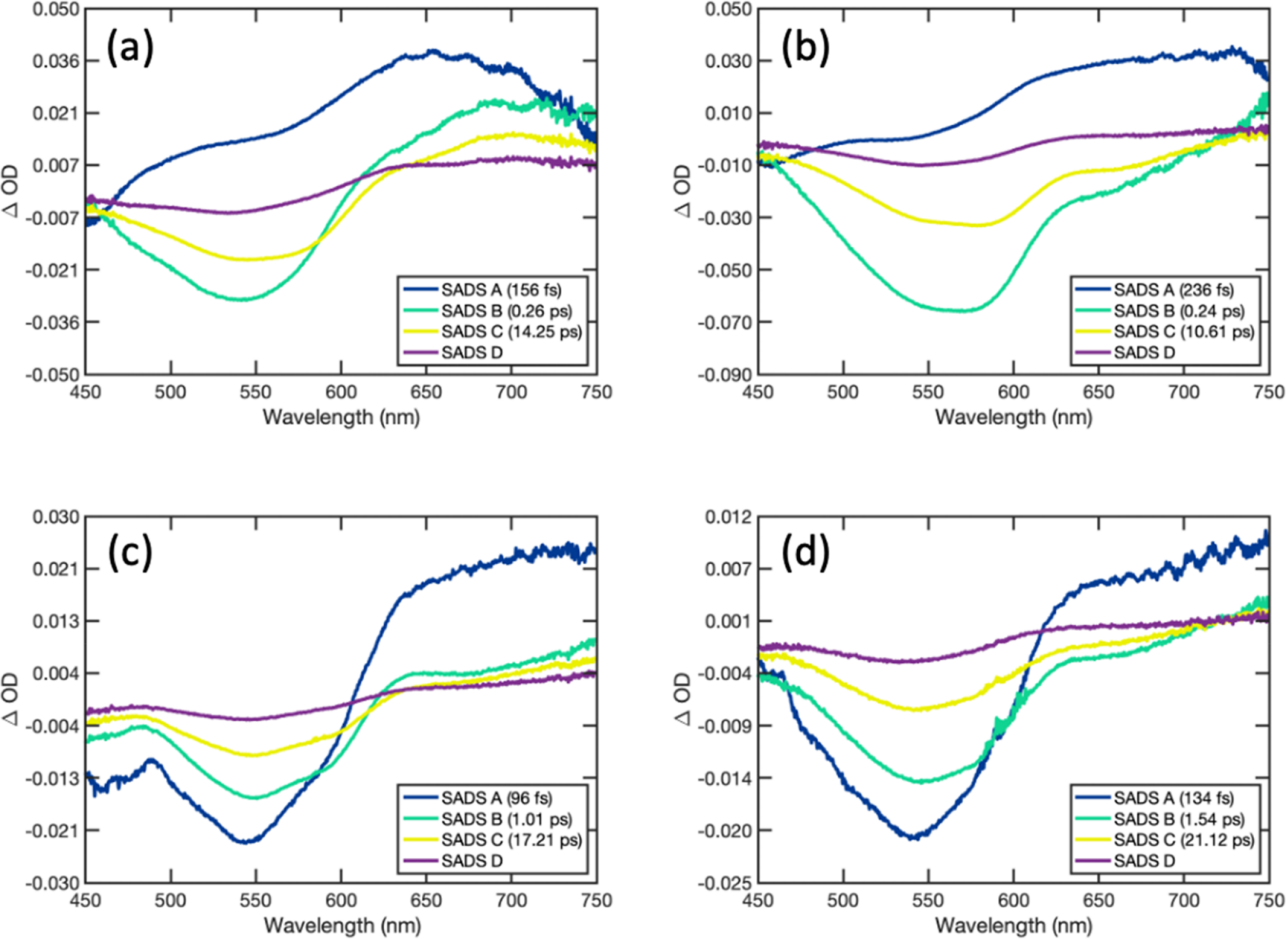
Species associated difference spectra (SADS) obtained from the global analysis of transient absorption spectra collected with PFNX:PTAK CPECs using the four-state kinetic interconversion model expressed by eq 5. (a) F4, (b) F2, (c) T1, and (d) T2. Lifetimes corresponding to each SADS are listed in Table S4.

We used global spectral analysis subject to sequential kinetic interconversion models to determine time scales on which spectral dynamics occur for all donor CPEs and PFNX:PTAK CPECs. We found that the best fitting agreement was obtained using a four-state sequential kinetic model, as expressed in eq 5, producing sensible species-associated difference spectra (SADS) that reflect the composition of excited species that give rise to TA signals for both donor CPEs and CPECs.
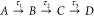
5SADS obtained from analysis of CPEC TA spectra is presented in Figure 6, with fitted transients at selected wavelengths presented in Figure S24. SADS obtained from the global analysis of TA data obtained with uncomplexed donors and corresponding fitted transients at select wavelengths are presented in Figures S25 and S26. Lifetimes corresponding to each SADS are presented in Tables S3 and S4 for complexes and donor CPEs, respectively.

For F2 and F4, the SADS capture the spectral evolution expected for donor–acceptor EET: In both cases, SADS A is dominated by the singlet excited-state absorption of the donor in the red (>550 nm) and stimulated emission below 500 nm; in both cases SADS A crosses ‘0’ at a wavelength similar to that observed in the donor CPE TA data. In contrast, SADS B is dominated by the PTAK bleach (475–625 nm). SADS B for F2 exhibits the PTAK stimulated emission (625–700 nm) observed via direct excitation of PTAK in CPECs (Figure 5). In contrast, the absorption that remains in this region for F4 most likely reflects uncomplexed regions of the donor in the corresponding solution. The transition from SADS B to C reflects spectral dynamics of PTAK excited by EET and, for F4, contributions from the spectral dynamics of uncomplexed regions of the donor. The differences in the PTAK bleach intensities in SADS B, C and D reflects fractions of PTAK excitons that deactivate on associated kinetic lifetimes summarized in Table S3. We previously demonstrated that charge separation is a viable quenching mechanism in PFNB:PTAK complexes probed in ultrafast experiments and is a likely origin of the long-lived bleach observed in these measurements.^7,10^

The SADS values obtained for T1 and T2 are somewhat different. In both cases, the PTAK bleach features dominantly in SADS A; this is consistent with either some direct excitation of the acceptor or very rapid (faster than our time resolution) EET from the donor. The former is plausible, given that the excitation wavelength used for these complexes, although close to the peak of the donor absorption feature, is also closer to that of PTAK. The latter is feasible, given the predicted and calculated rates of energy transfer *(e.g.*, Table 2 below). Nonetheless, a rapid decay of TA (>600 nm) and stimulated emission (<500 nm) signatures associated with the donor occurs on a time scale of less than 200 fs. The evolution of SADS B, C and D are largely similar across all complexes regardless of the donor, indicating that the associated spectral dynamics are largely attributed to excited PTAK.

**Table 2:** Measured EET Rates from Global Analysis along with Estimations Using the Förster Model

	PFNF4	PFNF2	PFNT1	PFNT2
EET rate (ps^–1^) (1/τ_EET_)	6.41	4.24	10.4	7.46
Forster ET rate (ps^–1^)	0.03	0.05	0.06	0.10

**Figure 7:**
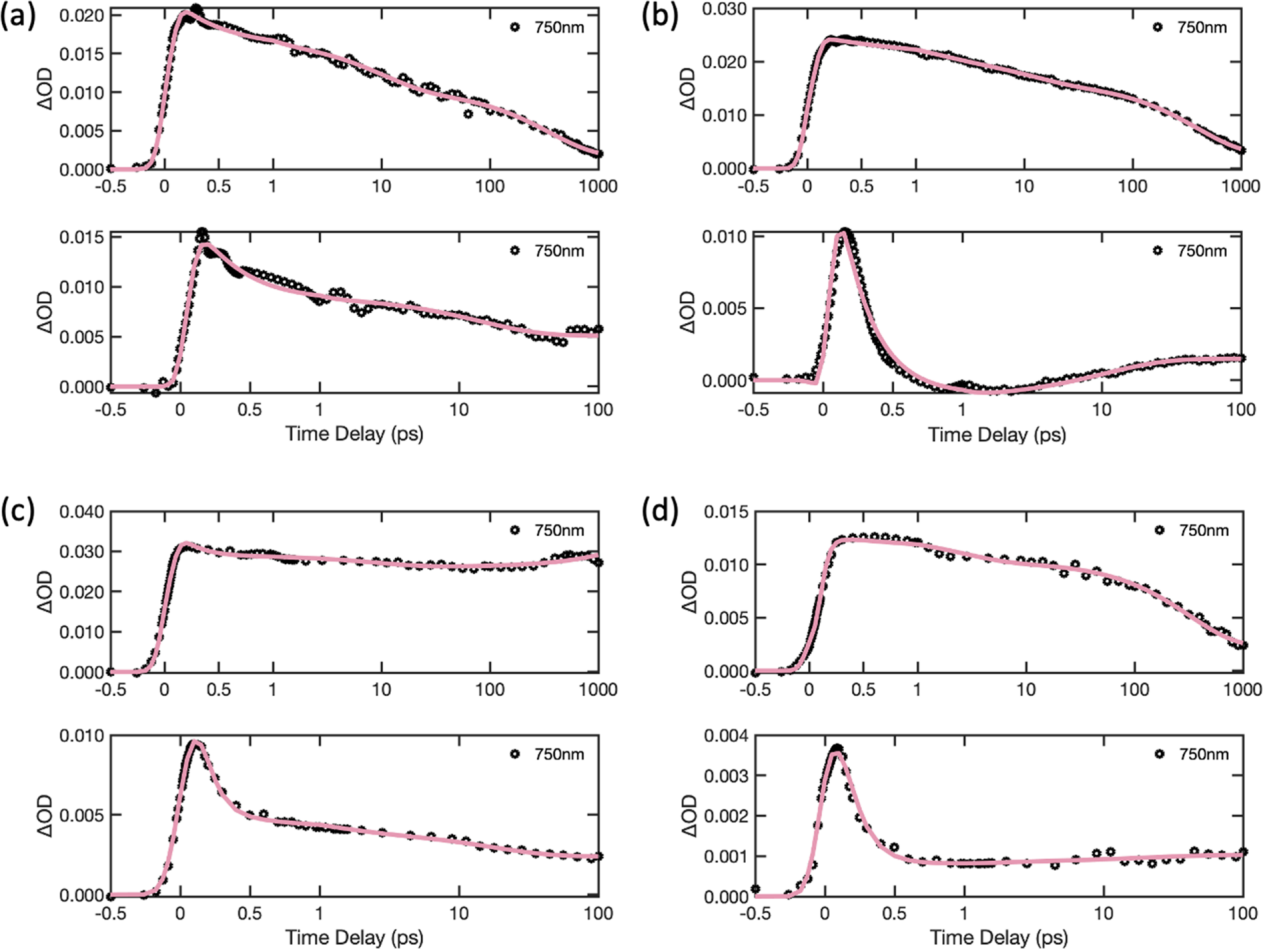
Comparison of transients obtained with donor CPEs and PFNX:PTAK CPECs by probing at 740 nm, within the region of photoinduced (transient) absorption of the donor: (a) F4, (b) F2, (c) T1, and (d) T2. Transients at more wavelengths for both donors and CPECs can be found in Figures S24 and S26.

Figure 7 compares the measured time dependences for donor CPEs and PFNX:PTAK complexes at a probe wavelength of 740 nm together with fits generated from global spectral analysis. These comparisons highlight the dramatic differences in excited donor CPE lifetimes upon complexation with PTAK that is consistent with rapid inter-CPE EET. Overall, the fits are of excellent quality, which allows us to extract quantitatively the EET time scales across the donor series. The EET rates (inverse EET times) from global analysis (τ_1_ from eq 5, values listed in Table S5) are shown in Table 2 along with EET rates calculated from the Förster model according to
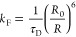
6where *τ*_D_ is the average radiative lifetime of the isolated donor. We note that the donor PL lifetime at room temperature was used for *τ*_D_, which is a lower bound given the presence of nonradiative relaxation pathways, and κ^2^ was taken to be unity for all CPECs. From global analysis we find that the shortest times (largest rates) are associated with T1 and T2 CPECs, followed by F4, F2 and B CPECs. We see that although the overall trend predicted by the Förster model is observed experimentally for F2, T1, and T2, it is clear that F4 stands out: its rate is comparable to T2 despite the large difference in spectral overlap. There is also an apparent disparity between the ratios of the rates. For example, under the assumption of a similar κ^2^, the ratio of *k*_F_ for T2 relative to F4 is ∼3.0, while the measured *k*_EET_ ratio is ∼1.2. Similarly, the predicted F2 to F4 *k*_F_ rate ratio is ∼1.3, while the measured *k*_EET_ ratio is ∼0.7.

### 3.3 Role of CPEC Conformation

**Figure 8:**
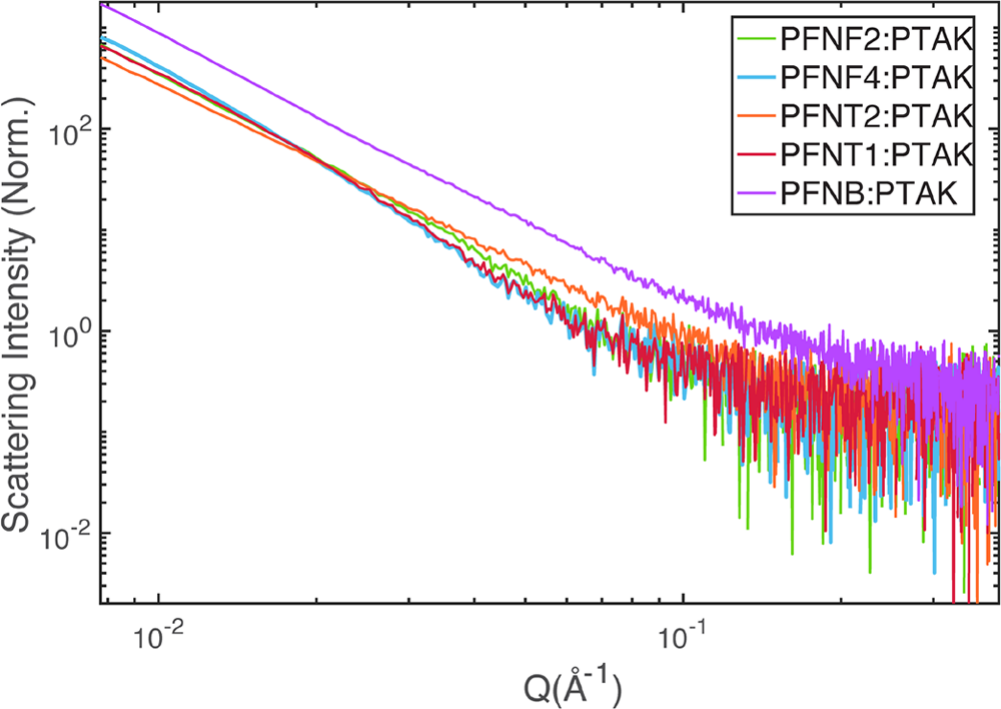
Azimuthally averaged small-angle X-ray scattering intensities as a function of the scattering vector length *Q* for all CPECs. All samples show monotonically decreasing intensities, with no visible Guinier plateau regions.

Since all donor CPE have similar ionic charge densities, it is tempting to assume that the CPEC conformations would be similar across the donor series. To determine whether this assumption is safe, we interrogated the CPEC conformation across the series using both structural and spectroscopic probes. Since CPECs are not expected to display long-range order, one of the more direct means of comparing the CPEC structure is to do so in reciprocal space by performing small-angle X-ray scattering (SAXS) measurements. We collected SAXS scattering images using a synchrotron X-ray source, and the isotropic SAXS intensity was then azimuthally averaged to produce reduced 1D scattering intensity curves as a function of scattering vector length *Q*. These results for all CPECs are shown in Figure 8.

The scattering intensity for all CPECs is monotonically decreasing, rather featureless, and reasonably similar in appearance. This suggests that, coarsely, the complexes have a similar microstructure. At low *Q*, the approximately linear slopes on a log–log plot suggest *Q* scaling with a comparable negative power-law exponent. However, closer inspection makes apparent the subtle differences between the curves. In particular, there appears to be an intermediate *Q* value (∼0.02 Å^–1^) at which the slope of the scattering intensity for the F2, F4, and T1 complexes undergoes a small decrease, which is not observed for B and T2 CPECs. This observation gives the first indication that the CPEC structures are similar but not identical across the donor series.

**Figure 9:**
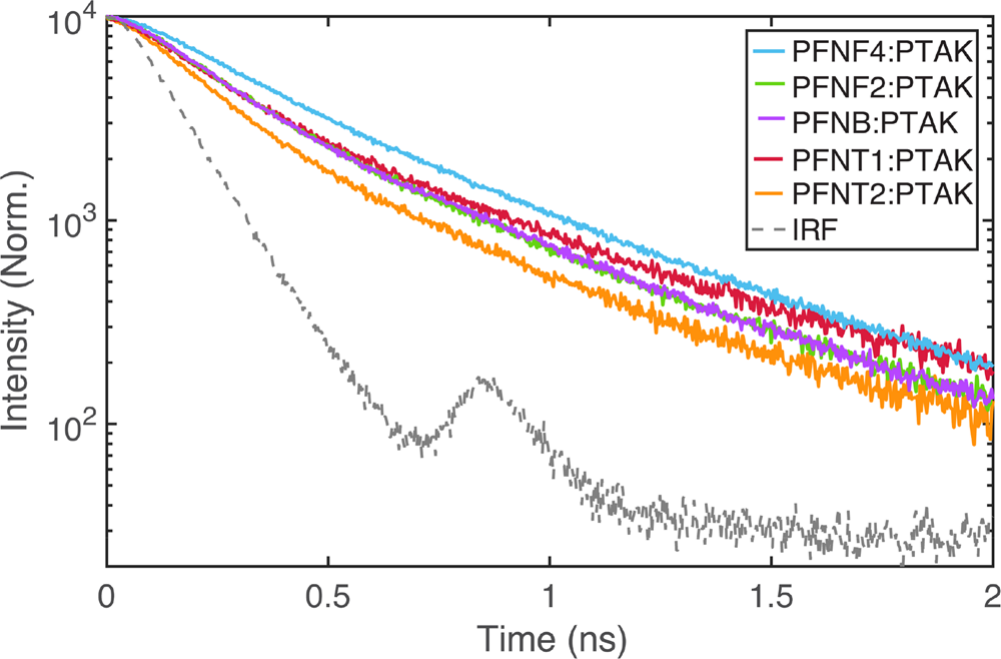
TRPL decays of selectively excited PTAK (600 nm) within each CPEC. The instrument response function is shown in gray.

With this indication in hand, we went on to interrogate the spectral characteristics of PTAK within the CPEC, since it is common to each complex and may thus act as a reporter for differences in complex conformation. Using time-correlated single-photon counting, we first collected time-resolved PL (TRPL) decays for each CPEC while exciting at 600 nm, where only PTAK absorbs. These results are shown in Figure 9. Each decay curve is well-described by a convolution of the instrument response function (IRF) with a sum of two exponential decays with the relatively short component being dominant. Fitting parameters following deconvolution of the IRF are shown in Table S5 of the Supporting Information. We find that PTAK in the T2 CPEC gives the shortest average PL lifetime (119 ps), while F4 gives the largest (150 ps). F2, B, and T1 show remarkably similar fast components, only showing subtle differences in the low-amplitude long component.

**Figure 10:**
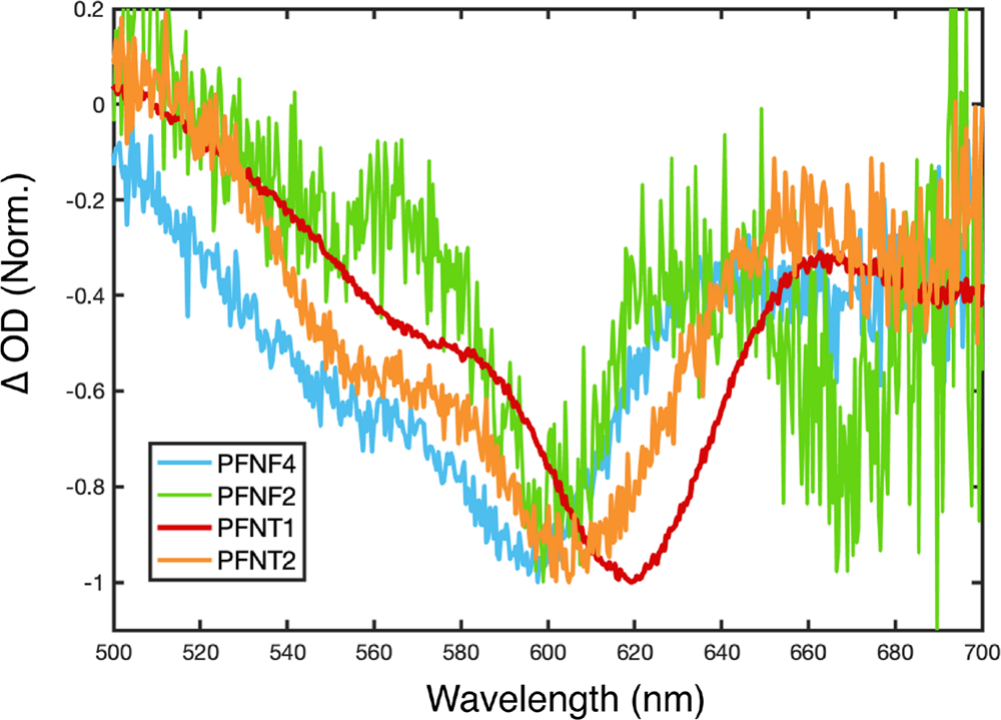
Normalized GSB spectra of CPECs at a 1 ps pump/probe delay excited at 600 nm. This wavelength corresponds to exclusive PTAK excitation. Small shifts in the absolute value of the peak are apparent across the donor series.

We then compared ultrafast GSB spectra of CPECs when PTAK is directly and exclusively excited within the complex at 600 nm. None of the donor CPEs absorb at this wavelength; thus, there is no possibility of EET. GSB spectra following 600-nm excitation at a pump–probe delay of 1 ps for all CPECs are shown in Figure 10. All of the PTAK GSB spectra within the CPEC across the donor CPE series are qualitatively similar to each other and qualitatively distinct from that of isolated PTAK (Figure 5). The similarity in PTAK GSB across different CPECs is consistent with PTAK vibronic 0–0/0–1 peak ratios in steady-state PL spectra of the complexes (Figure S19 of the Supporting Information), showing a J-aggregate-like vibronic ratio (0–0 intensity > 0–1 intensity). However, it is clear that the wavelength onsets of GSB signals on the red side exhibit minor shifts and some differences in the ratio of the 0–0 and 0–1 peak intensities, consistent with small differences in the onset of the PLE and OD spectra of the CPECs. Taken together, the SAXS, TRPL, and GSB following selective PTAK excitation all paint a similar picture: that the CPEC microstructure is qualitatively similar across the donor CPE series but exhibits quantitative differences as a function of monomer X.

## 4 Discussion

The steady-state PLE and ultrafast pump/probe data show that although we observe some qualitative agreement with the Förster model, it fails to account for the relative magnitude of *E*_rel_ and the EET rate for F4 as well as some of the rate ratios that we observe. This is not entirely surprising given the core assumption of the model, namely, that the Coulombic coupling between the exciton donor and acceptor could be described as a dipole–dipole interaction between *point* transition dipole moments. This assumption averages away all the details of the spatially extended transition densities of the two EET partners. As such, this model may become a progressively poorer approximation as the separation between the centers of mass of the two species becomes comparable to the lengths of their respective chromophores, *i.e*., the spatial extent of the excitonic wave functions. Indeed, it was shown previously that even for pigment molecules in natural light-harvesting antenna complexes, such as chlorophyll and carotenoid derivatives, the Förster approximation cannot always be justified.^42^

Division of *E*_rel_ by the spectral overlap integral (*E*_norm_) further suggests that excitonic coupling *V*_EX_ may differ substantially across the CPEC series. Specifically, it appears to be largest for F4 and comparable for F2 and T2. It is important to underscore once again that in calculating *E*_norm_ we implicitly assumed that the relaxation rate of donor excitons immediately following photoexcitation is faster than the EET rate. Given the relatively large EET rates that we measured, this assumption can be reasonably questioned. In the extreme case, the EET rate may be comparable or faster than the relaxation rate of the donor exciton. In such a case, division by *J* may no longer be justified. However, even in this case, the EET rate should scale like the square of *V*_EX_ multiplied by the density of final states at the exciton energy. We expect the latter to still be related to the magnitude of the PTAK absorption spectrum at that energy. The fact that the PTAK OD ratio for 400 nm relative to 360 nm (the wavelengths used to pump T2 and F4, respectively) is ∼2 while the ratio of *E*_rel_ and EET rates for T2 relative to F4 are ∼1.5 and ∼1.2, respectively, suggests that the same conclusion about the difference in *V*_EX_ for T2 vs F4 holds qualitatively as when *J* is divided out. As mentioned above, is likely preferentially underestimated for F4 compared to T2 given the deliberately limited PLE integration window.

The question that we aim to answer is, how can we understand the fact that *E*_rel_ and the EET rate for F4 are as large as they are given the fact that it has the smallest spectral overlap integral (or expectedly the smallest density of final acceptor states at the pump wavelength)? To help answer this question, we began by calculating several useful parameters of isolated donor CPE repeat units at the level of (time-dependent) DFT. Although the calculations are performed on a single repeat unit, we believe that this still provides a valuable comparison across the donor series treated on an equal footing. The fact that the calculation is carried out in vacuum means that the role of the environment on the repeat unit conformation is not taken into account. However, we expect that, to a good approximation, the intrinsic monomer–monomer interactions will dominate the electronic structure of the repeat unit in the complexed state. The results of these calculations are shown in Table 3. Two primary takeaways stand out. We find that the equilibrium torsion (dihedral) angle between the fluorene monomer and monomer X varies significantly (see Figure S28 of the Supporting Information for the explicit definition of the torsional angle). F4 has by far the largest angle (most twisted monomers), followed by F2 and B, while the thiophene-containing repeat units have the smallest angles. We find the opposite ordering of the transition dipole moments with T2 having the largest value and F4 the smallest.

**Table 3:** Repeat Unit Calculations Using DFT and TDDFT at the ωB97X-D3/def2-TZVPD Level of Theory^a^

	PFNF4	PFNF2	PFNB	PFNT1	PFNT2
Torsional angle (deg)	61.0	53.3	43.8	34.5	33.5
Transition dipole magnitude	2.31	2.47	2.63	3.01	3.58
Oscillator strength	0.640	0.728	0.817	1.021	1.378

a Torsional angles along the linkage bonds of copolymer units for PFNX are calculated for the ground-state equilibrium geometries. Transition dipole moments along the linkage axis and oscillator strengths are calculated for the lowest excited states.

**Figure 11:**
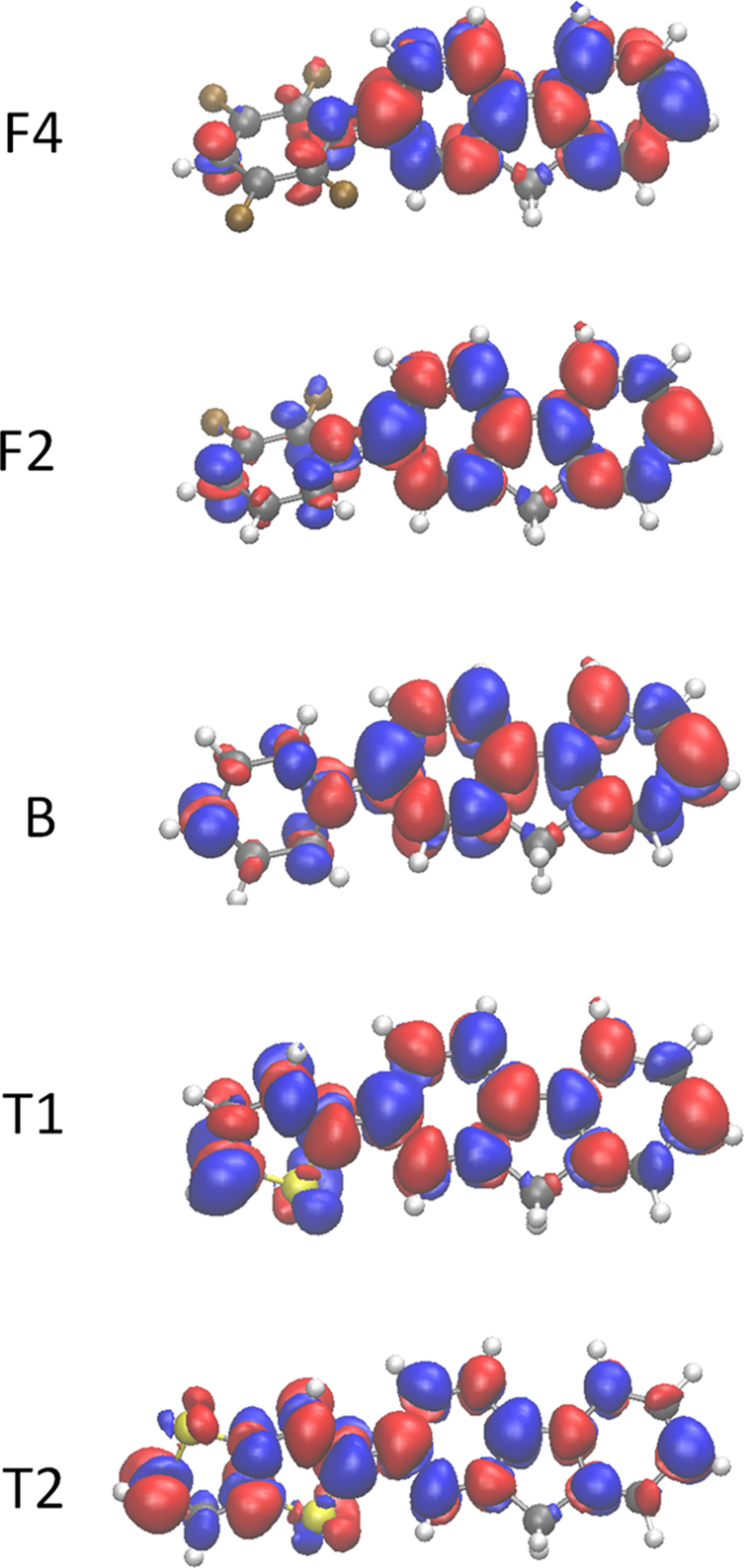
Transition density for each donor repeat unit on the electronic transition from the ground state to the lowest excited state. The blue and red colors correspond to negative and positive isosurfaces, respectively. The isovalue is 8 × 10^–4^ in a.u.

It is instructive to also visualize the transition densities of the repeat units, which are plotted in Figure 11. The transition densities on the fluorene monomer are similar for all. However, it is clear that the thiophene and thienothiophene monomers contain a significantly larger share of the transition density than the F2 and F4 repeat units, leading to a correspondingly larger degree of charge transfer across the two monomers (see Figure S29 of the Supporting Information for charge difference densities between the ground states and the lowest excited states of PFNF4, PFNF2, PFNT1, and PFNT2). This suggests that over the length of a donor polymer chromophore the transition density for F2 and F4 will display significant position-dependent variations in magnitude, whereas for T1 and T2 the transition density will depend significantly more weakly on the position along the chromophore.

What are the implications of these calculations for EET within CPECs? We first consider the influence of the torsion angle. In conjugated polymers, the excitonic wave function can be described as a product of (i) a center-of-mass (envelope) wave function that dictates the spatial extent of the exciton and (ii) a relative wave function the describes the probability to observe the electron and the hole at a given separation.^43,44^ It is reasonable to expect that as the average torsion angle increases, the center-of-mass excitonic wave function will become dynamically more localized, leading to a smaller mean exciton extent. Thus, we expect that F4 will have the smallest exciton radius. To appreciate the significance of this expectation for EET, it is instructive to consider the line-dipole approximation, which is a substantial improvement from the Förster model for conjugated polymers. Within this approximation, the electronic coupling between two proximal conjugated polymer chains is given by a sum of pairwise interactions between transition dipole moments of each monomer on the first chain and that of each monomer on the second chain. The specific monomer–monomer interaction is still described by the dipole term in the multipole expansion of the full Coulomb interaction. We doubt that even this approximation is an excellent one given the relatively small separation between the donor and the acceptor within the CPEC. The approximate condition for its validity is that the length of the repeat unit be significantly smaller than the center-to-center separation between the two parallel conjugated-polymer chains, which is not expected to be completely fulfilled in our CPECs. A more accurate way of describing the excitonic coupling is the transition-density cube method, but it is computationally taxing and does not readily yield qualitative insight.^45^ In contrast, the line-dipole approximation provides significant qualitative insight that can aid the interpretation of our results.

Barford has shown analytically that for parallel conjugated polymer chains within the line-dipole approximation, the excitonic coupling is a decreasing function of chromophore length, *i.e*., the length over which the excitonic wave function is coherently delocalized.^46^ For identical chains, , where *L* is the chromphore length, and *p* ranges from 1 to ∼2 depending on the assumptions made about the functional form of the center-of-mass excitonic wave function. In other words, perhaps somewhat counterintuitively, as the exciton radius *decreases*, the excitonic coupling is expected to *increase*. Barford’s analytical results are supported by quantum-chemical calculations, which show that the coupling increases as either the donor or the acceptor chromophore length decreases as soon as the chromophore length extends beyond a couple of repeat units.^47,48^

These analytical and computational results allow us to propose an explanation for why F4 stands out in the magnitude of its *E*_rel_ and its relatively large EET rate given its relatively large bandgap and thus poor spectral overlap with PTAK. Its largest torsion angle likely leads to the smallest extent of the center-of-mass excitonic wave function. Moreover, the red edge of the OD and the PLE spectra for the F4 CPEC (blue curves in Figure 2A and B, respectively) shows that the PTAK region is most blue-shifted compared to the rest of the CPECs. Within the particle-in-a-box approximation, this suggests that the spatial extent of the PTAK exciton is also the smallest for the F4 CPEC. Taken together, we believe that these observations are largely responsible for the apparently disproportionately large excitonic coupling in the F4 CPEC. The fact that *E*_norm_ is comparable for F2 and T2 can be rationalized by the fact that, although the torsion angle for F2 is larger than that of T2, the transition dipole moment of the T2 repeat unit is larger than that of the F2 repeat unit. We note that at the moment it is not entirely clear what the source of the disagreement is between the relative position of *E*_norm_ for T1 within the donor series and its measured EET rate.

Although we believe that the above considerations are largely responsible for our EET observations, there are two additional considerations that are worth mentioning. First, it is possible that the larger spatial variation in the transition density magnitude along the chromophore for F2 and F4 compared to T1 and T2 may lead to further differences. We may anticipate two potential effects: (i) The lateral shifts between monomers in parallel chains may lead to variations in the interchain excitonic coupling.^14,49^ The somewhat smaller linear charge density of T2 could be one source of an average lateral shift between donor and acceptor excitonic wave functions compared to other donor CPEs. (ii) As the degree of charge transfer between the fluorene monomer and the variable monomer increases, the relative wave function of the exciton may become a mixture of neutral Frenkel-type and charge-transfer-type states.^50^

Our results show that although the average CPEC structures appear similar, they are not identical. Differences in structure could lead to subtle variations in the relative orientation between the donor and acceptor chromophores, which would modify the EET rate. For example, in addition to electrostatic interactions between the polyanion and the polycation, the fluorinated benzene monomer in F4 may participate in anion-π interactions with the carboxylate group at the terminus of the PTAK side chain. Moreover, it is conceivable that there may be some degree of π-stacking between the donor and acceptor backbones, although we believe the extent of such interactions would likely be limited by the geometry of the electrostatic binding. Nevertheless, π-stacking would lead to a modification of the exciton transfer integral.^46^

In closing, we note that we observe a steady increase in EET efficiency of the CPEC for all donor CPEs as measured by PLE spectroscopy on the days to weeks time scale, as shown in Figure S27 of the Supporting Information. We attribute this increase primarily to the condensation of multiple chains onto the complex, leading to an increase in the local concentration of donor and acceptor chromophores.^51^ This is consistent with the appearance of a measurable sample turbidity after approximately 1 week, in contrast to fresh CPEC solutions. In the very long time limit, we expect to observe the onset of macroscopic phase separation. Although EET efficiencies increase for all donor CPEs, the increases for F4 and F2 are much more dramatic relative to the rest of the donor polymers. Future work will focus on elucidating the details of this slow evolution of the CPEC assembly.

## 5 Conclusion

In this report, we synthesized two subfamilies of exciton-donor alternating copolymers consisting of ionic fluorene monomers and alternating thiophene-based and fluorine-substituted phenyl-based monomers while keeping the linear ionic charge density fixed within a narrow range. We then interrogated their EET characteristics and found that although the Förster model is qualitatively consistent with *some* of the EET trends that we observe, it fails to qualitatively account for the ordering of the entire donor CPE series and to apparently quantitatively account for the ratios of relative EET efficiencies and EET rates. Specifically, we find that the donor CPE containing a tetrafluoro-substituted phenyl comonomer displays a disproportionately large EET rate and relative EET efficiency. We largely rationalize this finding by considering how the excitonic coupling is expected to scale with the mean exciton delocalization length.

Our results indicate that although the positions of steady-state donor PL and acceptor OD spectra provide a reasonable starting point for a rough expectation of EET efficiency within a CPEC, given the relatively small donor/acceptor separations involved, the influence of precise chemical structure of the CPE backbone on the excitonic coupling cannot be overlooked. This has implications for the choice of a particular donor/acceptor CPE pair, if the goal is to maximize the EET rate in a particular spectral window.

We envision CPECs functioning as key exciton-transferring antenna components in an overarching soft-matter-based, water-based light-harvesting material. In such materials, different rate processes must often be carefully balanced to ensure optimal energy conversion efficiencies. Knowing how subtle modifications to the chemical structure of a CPE backbone alter EET is a key first step toward engineering CPECs to serve as rapid relays for exciton energy transport.

## Supporting Information Available

Synthesis details, purchased synthetic materials, ^1^H NMR characterization of all CPEs, high-temperature size exclusion chromatography (HT-SEC), fits of photoluminescence excitation spectra, time-resolved photoluminescence lifetimes of all CPEs and CPECs, transient absorption false color plots, transient absorption waterfall plots, species-associated difference spectra (SADS), global analysis single-wavelength slices, and DFT calculations of torsional angles and charge difference density plots of repeat units (PDF)
