# Supplementary material for: Exciton Transfer Between Extended Electronic States in Conjugated Inter-Polyelectrolyte Complexes
Source: ACS Appl Mater Interfaces. 2024 Jan 30;16(16):19995–20010. doi: 10.1021/acsami.3c14657 (PMC11056932; doi:10.1021/acsami.3c14657)
Supplement: Supplementary file 1 — am3c14657_si_001.pdf [file am3c14657_si_001.pdf]

Supporting Information

Exciton Transfer Between Extended Electronic States in Conjugated Inter-Polyelectrolyte Complexes

Rachael Richards,[1≠] Yuqi Song,[2≠] Luke O'Connor,[2] Xiao Wang,[1] Eric A. Dailing,[3] Arthur E. Bragg,[2]* Alexander L. Ayzner[1]*

[1] Department of Chemistry and Biochemistry, University of California Santa Cruz, Santa Cruz, CA, USA

[2] Department of Chemistry, Johns Hopkins University, Baltimore, MD, USA

[3] The Molecular Foundry, Lawrence Berkeley National Laboratory, Berkeley, CA, USA

* aayzner@ucsc.edu
* artbragg@jhu.edu

S1. Synthesis

Materials: The reagents and materials utilized in this study were obtained directly from the specified distributors without any further modifications: 2,7-dibromofluorene and 4-toluenesulfonyl chloride obtained from Oakwood Chemical. Poly[3-(potassium-4-butanoate)thiophene-2,5-diyl] regioregular was obtained from Rieke Metals. Monomers 3,6-Dibromothieno[3,2-b]thiophene (98.0%) and 1,4-Dibromo-2,3-difluorobenzene, (98.0%) were purchased from TCI America. Monomer 1,4-Dibromotetrafluorobenzene (99%) was purchased from Sigma Aldrich. Monomer 1,4-Dibromobenzene (97.0%) was purchased from Spectrum Laboratory Products Inc. SlideALyzer Dialysis Flasks, 10K MWCO, 250mL obtained from Fisher Scientific Company LLC. Bis(pinacolato)diboron (98%) and 1,4-dioxane (anhydrous, 99.8%) were obtained from Alfa Aesar. Tetraethylammonium bromide (100%) was obtained from Chem-Impex International Inc. Palladium catalyst [1,1'-bis(diphenylphosphino)ferrocene] dichloropalladium (II), dimethyl aminopropyl chloride hydrochloride (96%), were obtained from Sigma-Aldrich. Methyl iodide (99.5%), tetrahydrofuran (HPLC grade), and dichloromethane (99.5%) were obtained from Spectrum Laboratory Products. Sodium hydroxide, sodium hydroxide solution (50% w/w), potassium

carbonate (anhydrous, 99.7%), triethylamine (99%), diethyl ether (99%), methanol (99.9%), acetone (HPLC grade), chloroform (99.9%), ethyl acetate (99.5%), hexanes (98.5%), and potassium acetate (>99%) were obtained from Fisher Scientific Company LLC. Deuterated solvents CDCl3 (D 99.8%) and D2O (D 99.9%) were both purchased from Cambridge Isotope Laboratories. Nuclear magnetic resonance (*NMR*) spectra of the polymers were collected on a Bruker Avance III HD 4 channel 800 MHz NMR with a cryoprobe. Due to solubility restrictions of the conjugated polymers, ^{13}C NMR was not obtained. Below are the ^{1}H NMR spectra of all materials synthesized.

1.1 Synthesis of Monomer FN:

Synthesis of *(2,7-dibromo-9,9-bis(3'-(N,N-dimethyl-amino)-propyl)- fluorene)* To a clean, dried, 100 mL two neck round bottom flask, a Teflon coated stir bar, dimethyl sulfoxide (*DMSO*, 30.9 mL, 434.6 mmol), 2,7- dibromofluorene (*F*, 2 g, 6.2 mmol), tetrabutylammonium bromide (*TBAB*, 39.8 mg, 0.12 mmol), and 4 mL of a 50 wt. % aqueous sodium hydroxide solution (*50 wt. % aq. NaOH*, 4 mL, 154.3 mmol) was added under an atmosphere of N_2(g). An additional aliquot of DMSO (10.5 mL, 145.1 mmol) was added to the reaction flask, followed by dimethyl aminopropyl chloride hydrochloride salt (*DAPCl*, 2.6 g, 16.4 mmol). The reaction was stirred and heated at 60 °C for 12 hours. Reaction progress was monitored by thin-layer chromatography (*TLC*). DI H_2O (40 mL, 2.216 mmol) was added to the reaction flask to dissolve precipitated salts as well as to solvate DMSO. The product (FN) was extracted from the wet DMSO layer with diethyl ether (*Et_2O*, 8 x 25 mL), washed with a 10 wt. % aqueous NaOH (2 x 50 mL). The organic layer was washed with DI H_2O (3 x 50 mL), followed by a brine wash (1 x 50 mL), and then dried over Na_2SO4. Concentration of the anhydrous organic layer under reduced pressure lead to crude solid which was purified with a silica gel column (Hexanes: Ethyl Acetate: Triethylamine,

49:49:2) to obtain FN (72% yield).

1.2 Synthesis of Monomer FNB

Synthesis of *(2,7-diboryl pinacol ester-9,9-bis(3'-(N,N-dimethyl- amino)-propyl)-fluorene)* To a clean, dried, 100 mL two neck round bottom flask, a Teflon coated stir bar, dimethylformamide (*DMF*, 39 mL, 505.8 mmol), FN (1 g, 2.0 mmol), bis(pinacolato)diboron (*B_2Pin_2*, 2.26 g, 8.9 mmol), potassium acetate (*KOAc*, 3.53 g, 17.8 mmol), [1,1'-Bis(diphenyl- phosphino)ferrocene]dichloro-palladium(II) (*$Pd(dppf)Cl_2$*, 0.296 g, 0.40 mmol) were added under an atmosphere of N_2(g). The contents of the reaction were stirred and heated at 80 °C for 24 hours. Reaction progress was monitored by TLC. Upon completion, the reaction was concentrated to dryness, and the crude solid was extracted with hot HPLC-grade hexanes (7 x 100 mL). The combined hexanes layer was filtered, concentrated to dryness, reextracted with hot hexanes, and re- concentrated to dryness. Acetone was used to extract the product from the re-dried hexanes layer and was allowed to

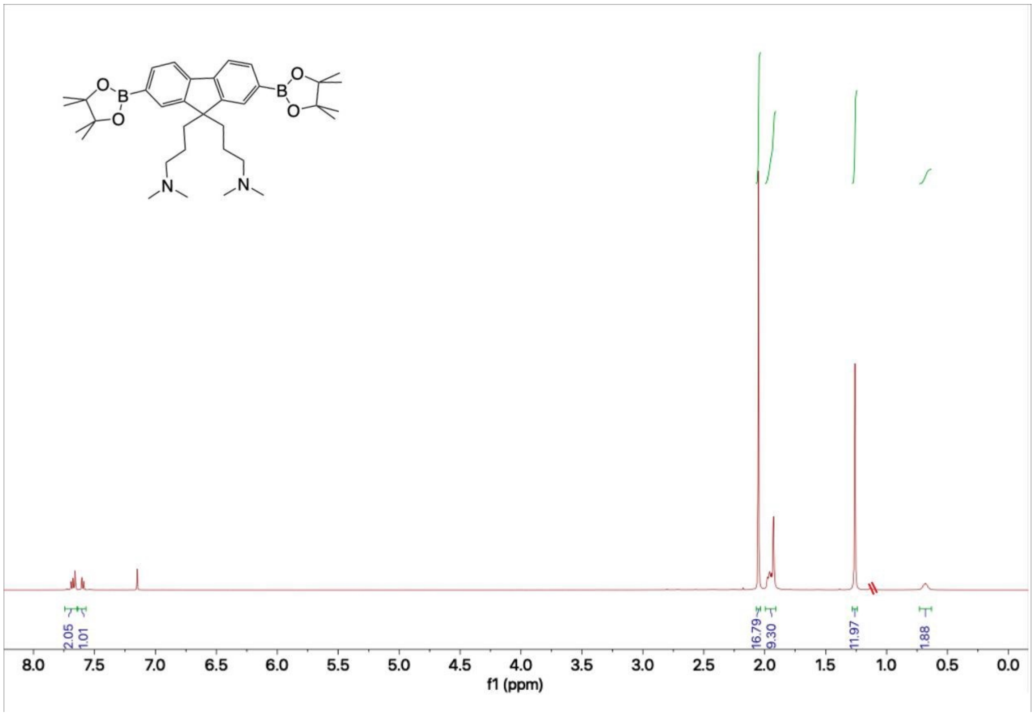

Figure S1. FNB 1H 500 MHz NMR in $CDCl_3$.

crystallize out of the solution as an off-white solid. The crystals of FNB were collected via filtration and washed with a minimal amount of cold acetone to obtain FNB (82.5% yield).

1.3 Synthesis of Neutral Polymer nPFNT1

Polymerization of *3,3'-(2-methyl-7-(5-methylthiophen-2- yl)-9H-fluorene-9,9-diyl)bis(N,N-dimethylpropan-1-amine)* clean pressured vial, 2,5- dibromothiophene (350 mg, 1 mmol), FNB (500 mg, 0.84 mmol), potassium carbonate (K_2CO_3, 1.25 g, 9.6 mmol), 1,4- dioxane (*Dioxane*, 8 mL, 90.4 mmol), DI H_2O (7.0 mL, 277.0 mmol), and $Pd(dppf)Cl_2$ (4 mg, 0.005 mmol) were added. The entire reaction vessel, including the solution was flushed with N_2(g). The pressured vial was placed into a silicon oil bath, stirred, and heated at 100 °C for 48 hours. To stop the reaction, the stirring function was turned off and the polymer in the dioxane layer was pipetted into DI H2O to induce precipitation of nPFNT1, decanted and vacuum filtrated.

1.4 Synthesis Conjugated Polyelectrolyte PFNT1

Conjugated polyelectrolyte 3,3'-(2-methyl-7-(5- methylthiophen-2-yl)-9*H*-fluorene-9,9-diyl) bis(*N*,*N*,*N*-trimethylpropan-1-aminium). In-situ quaternization of nPFNT1 was done with the addition of iodomethane (MeI, 4 mL, 62 mmol) directly to the remaining dioxane layer containing nPFNT1 and was left to react for 2 hours before the addition of DI H_2O, to assist in the dissolution of the polymer. The reaction was then heated to 50 °C for 5 days and aliquots of DI H_2O were systematically added until there was no more headspace in the 250mL pressured vial and the polymer was well dissolved. The solution was then dialyzed via the Thermo Scientific™ Slide-A-Lyzer Dialysis Cassette (10,000 MWCO) submerged in a vat of DI H_2O. The DI H_2O was exchanged with fresh DI H_2O every day for 3 days. After 3 days, the dialyzed solution of PFNT1 was concentrated under reduced pressure, filtered, transferred into multiple 25 mL Falcon tubes, and lyophilized to yield PFNT1 as a rusty-brown solid (71.2% yield). ^{1}H NMR: δ 2.07, 2.13, 2.22, 2.28, 2.85, 3.01, 3.07, 3.13, 7.03,

7.20, 7.47, 7.34, 7.40, 7.51,7.99 7.57, 7.59, 7.70, 7.81, 7.90, 8.04.

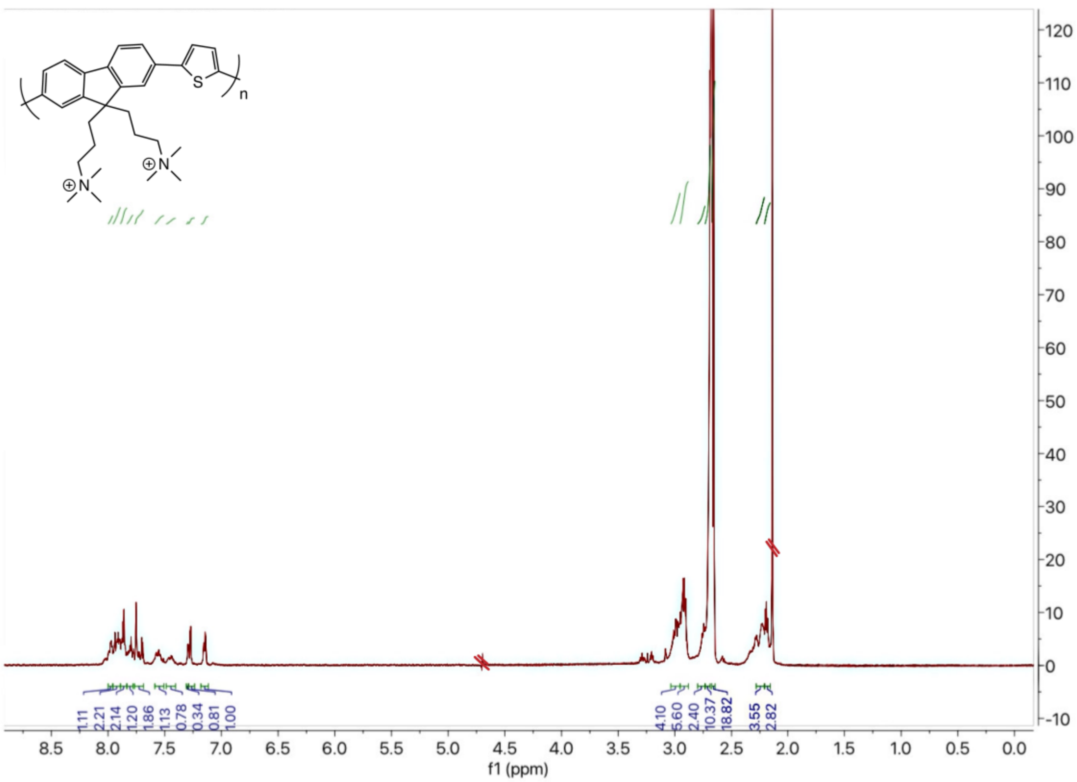

Figure S2: PFNT1 ^{1}H 800 MHz NMR in D_2O with a small water suppression peak ~4.7ppm and trace amounts of acetone.

1.5 Synthesis of Neutral Polymer nPFNT2

Polymerization of *3,3'-(2-methyl-7-(5-methylthieno[3,2- b]thiophen-2-yl)-9H-fluorene-9,9-diyl)bis(N,N-dimethylpropan-1-amine).* To a clean pressured vial, 2,5-dibromothieno[3,2-b]thiophene (300 mg, 1 mmol), FNB (500 mg, 0.84 mmol), potassium carbonate (K_2CO_3, 1.25 g, 9.6 mmol), 1,4- dioxane (*Dioxane*, 8 mL, 90.4 mmol), DI H_2O (7.0 mL, 277.0 mmol), and Pd(dppf)Cl_2 (4 mg, 0.005 mmol) were added. The entire reaction vessel, including the solution was flushed with N_2(g). The pressured vial was placed into a silicon oil bath, stirred, and heated at 100 °C for 48 hours. To stop the reaction, the stirring function was turned off and the polymer in the dioxane layer was pipetted into DI H_2O to

induce precipitation of nPFNT2, decanted and vacuum filtrated to dry.

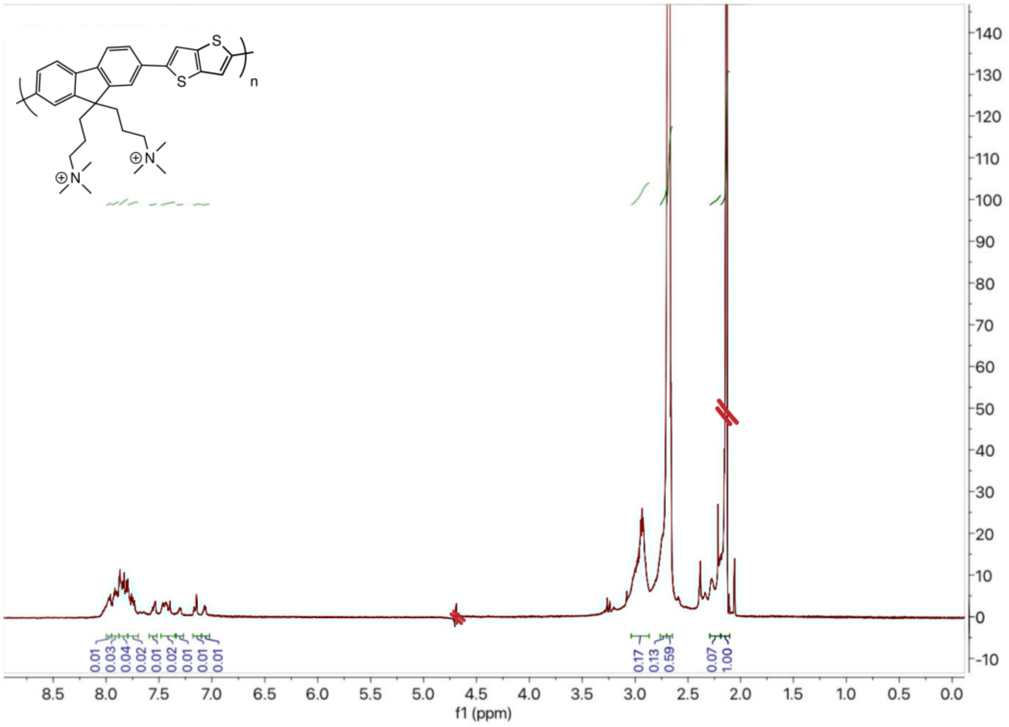

Figure S3: PFNT2 ^{1}H 800 MHz NMR in D_2O with a small water suppression peak ~4.7ppm and trace amounts of acetone

1.6 Synthesis Conjugated Polyelectrolyte PFNT2

Conjugated polyelectrolyte *3-(2-methyl-7-(5- methylthieno[3,2-b]thiophen-2-yl)-9-(3-(trimethylammonio)propyl)-9H-fluoren-9-yl)propyl)-l4- azaneyl)methylium*. In-situ quaternization of nPFNT2 was done with the addition of iodomethane (MeI, 4 mL, 62 mmol) directly to the remaining dioxane layer containing nPFNT2 and was left to react for 2 hours before the addition of DI H_2O, to assist in the dissolution of the polymer. The reaction was then heated to 50 °C for 5 days and aliquots of DI H_2O were systematically added until there was no more headspace in the 250mL pressured vial and the polymer was well dissolved. The solution was then dialyzed via the Thermo Scientific™ Slide-A-Lyzer Dialysis Cassette (10,000 MWCO) submerged in a vat of DI H_2O. The DI H_2O was exchanged with fresh DI

H_2O every day for 3 days. After 3 days, the dialyzed solution of PFNT2 was concentrated under reduced pressure, filtered, transferred into multiple 25 mL Falcon tubes, and lyophilized to yield PFNT2 as a rusty- brown solid (65.03% yield). 1H NMR: δ 2.07, 2.13, 2.16, 2.24, 2.85, 3.01-3.13, 7.05, 7.10, 7.30-7.46, 7.38, 7.57, 7.71, 7.76, 7.81, 7.90, 7.94, 8.01, 8.09.

1.7 Synthesis of Neutral Polymer nPFNF2

Polymerization of *3,3'-(2-(2,3-difluoro-4-methylphenyl)-7- methyl-9H-fluorene-9,9-diyl)bis(N,N-dimethylpropan-1-amine).* To a clean pressured vial 1,4- dibromo-2,3-difluorobenzene (300 mg, 0.98 mmol), FNB (500 mg, 0.84 mmol), potassium carbonate (K_2CO_3, 1.25 g, 9.6 mmol), 1,4- dioxane (*Dioxane*, 8 mL, 90.4 mmol), DI H_2O (7.0 mL, 277.0 mmol), and Pd(dppf)Cl_2 (4 mg, 0.005 mmol) were added. The entire reaction vessel, including the solution was flushed with N_2(g). The pressured vial was placed into a silicon oil bath, stirred, and heated at 100 °C for 48 hours. To stop the reaction, the stirring function was turned off and the polymer in the dioxane layer was pipetted into DI H_2O to induce precipitation of nPFNF2, decanted and vacuum filtrated to dry.

1.8 Synthesis Conjugated Polyelectrolyte PFNF2

Conjugated polyelectrolyte *3,3'-(2-(2,3-difluoro-4- methylphenyl)-7-methyl-9H-fluorene-9,9-diyl)bis(N,N,N-trimethylpropan-1-aminium).* In-situ quaternization of nPFNF2 was done with the addition of iodomethane (MeI, 4 mL, 62 mmol) directly to the remaining dioxane layer containing nPFNF2 and was left to react for 2 hours before the addition of DI H2O, to assist in the dissolution of the polymer. The reaction was then heated to 50 °C for 5 days and aliquots of DI H2O were systematically added until there was no more headspace in the 250mL pressured vial and the polymer was well dissolved. The solution was then dialyzed via the Thermo Scientific™ Slide-A-Lyzer Dialysis Cassette (10,000 MWCO) submerged in a vat of DI H2O. The DI H2O was exchanged with fresh DI H2O every day for 3 days. After

3 days, the dialyzed solution of PFNF2 was concentrated under reduced pressure, filtered, transferred into multiple 25 mL Falcon tubes, and lyophilized to yield PFNF2 as a light grey solid (79.08% yield). 1H NMR: δ 2.07, 2.10, 2.13, 2.15, 2.28, 2.33, 2.33, 2.85, 3.01, 3.07, 3.18, 7.48, 7.58, 7.60, 7.63, 7.73- 7.89, 8.13, 8.21.

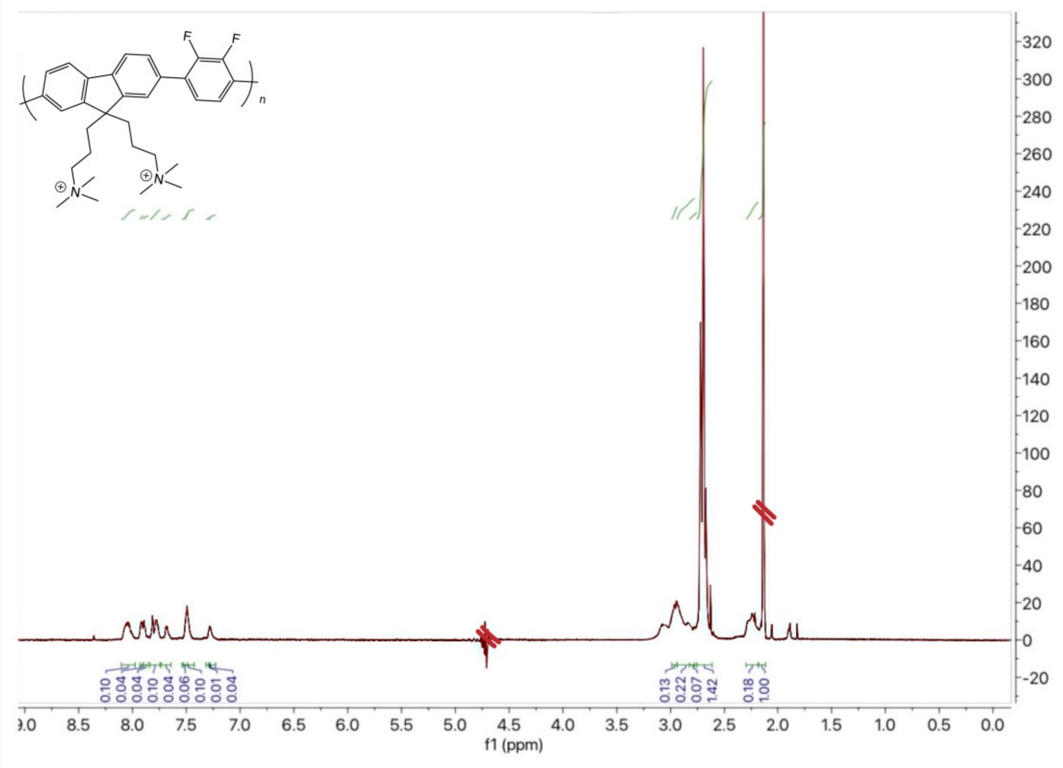

Figure S4: PFNF2 ^{1}H 800 MHz NMR in D_2O with a small water suppression peak ~4.7ppm and trace amounts of acetone.

1.9 Synthesis of Neutral Polymer nPFNB

Polymerization of *3,3'-(2-methyl-7-(p-tolyl)-9H-fluorene-9,9-diyl)bis(N,N-dimethylpropan-1-amine).* To a clean pressured vial 1,4-dibromobenzene (300 mg, 0.74 mmol), FNB (500 mg, 0.84 mmol), potassium carbonate

(K_2CO_3, 1.25 g, 9.6 mmol), 1,4- dioxane (*Dioxane*, 8 mL, 90.4 mmol), DI H_2O (7.0 mL, 277.0 mmol), and $Pd(dppf)Cl_2$ (4 mg, 0.005 mmol) were added. The entire reaction vessel, including the solution was flushed with N_2(g). The pressured vial was placed into a silicon

oil bath, stirred, and heated at 100 °C for 48 hours. To stop the reaction, the stirring function was turned off and the polymer in the dioxane layer was pipetted into DI H_2O to induce precipitation of nPFNB, decanted and vacuum filtrated to dry.

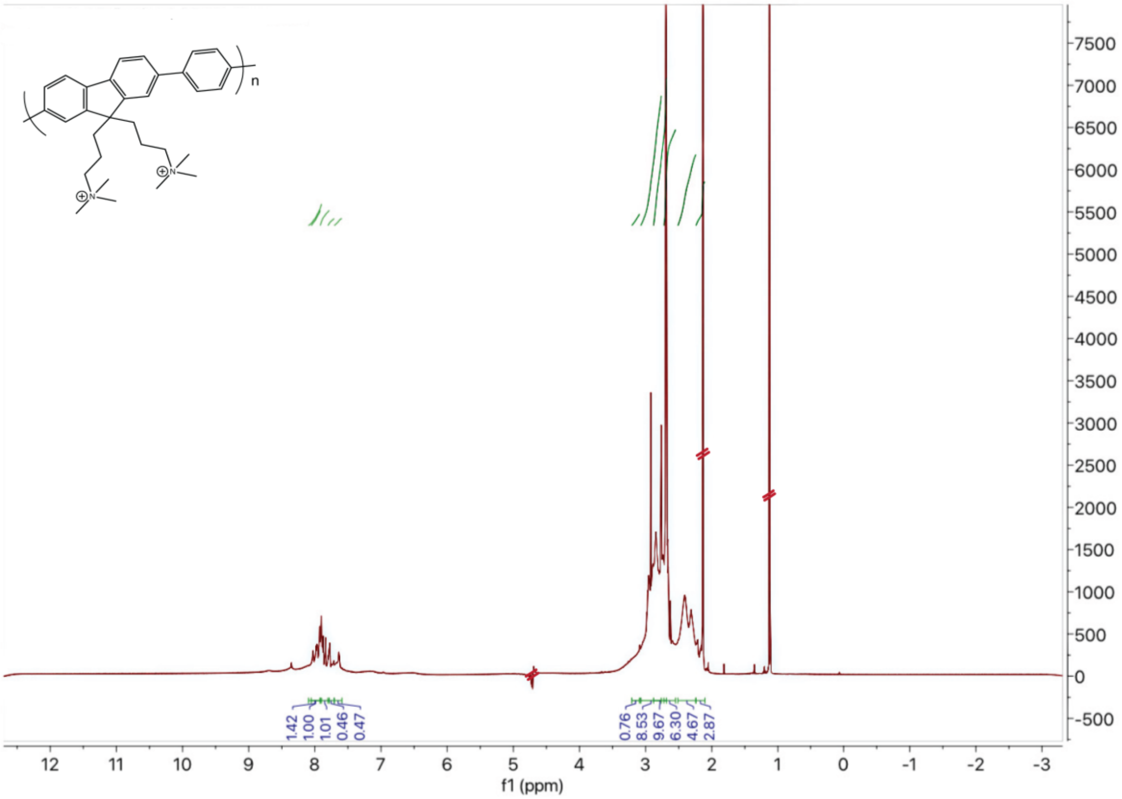

Figure S5: PFNB ^1H 800 MHz NMR in D_2O with a small water suppression peak ~4.7ppm and trace amounts of acetone and hexanes. Broadening of baseline observed around 6.5-7.3ppm due to low solubility.

1.10 Synthesis Conjugated Polyelectrolyte PFNB

Conjugated polyelectrolyte *3,3'-(2-methyl-7-(p-tolyl)-9H-fluorene-9,9-diyl)bis(N,N,N-trimethylpropan-1-aminium)*. In-situ quaternization of nPFNB was done with the addition of iodomethane (MeI, 4 mL, 62 mmol) directly to the remaining dioxane layer containing nPFNF2 and was left to react for 2 hours before the addition of DI H2O, to assist in the dissolution of the polymer. The reaction was then heated to 50 °C for 5 days and aliquots of DI H2O were systematically added until there was no more headspace in the 250mL

pressured vial and the polymer was well dissolved. The solution was then dialyzed via the Thermo Scientific™ Slide-A-Lyzer Dialysis Cassette (10,000 MWCO) submerged in a vat of DI H2O. The DI H2O was exchanged with fresh DI H2O every day for 3 days. After 3 days, the dialyzed solution of PFNB was concentrated under reduced pressure, filtered, transferred into multiple 25 mL Falcon tubes, and lyophilized to yield PFNB as a light yellow solid (61.41% yield). 1H NMR: δ 2.07, 2.10, 2.13, 2.15, 2.28, 2.33, 2.33, 2.85, 3.01, 3.07, 3.18, 7.48, 7.58, 7.60, 7.63, 7.73- 7.89, 8.13, 8.21.

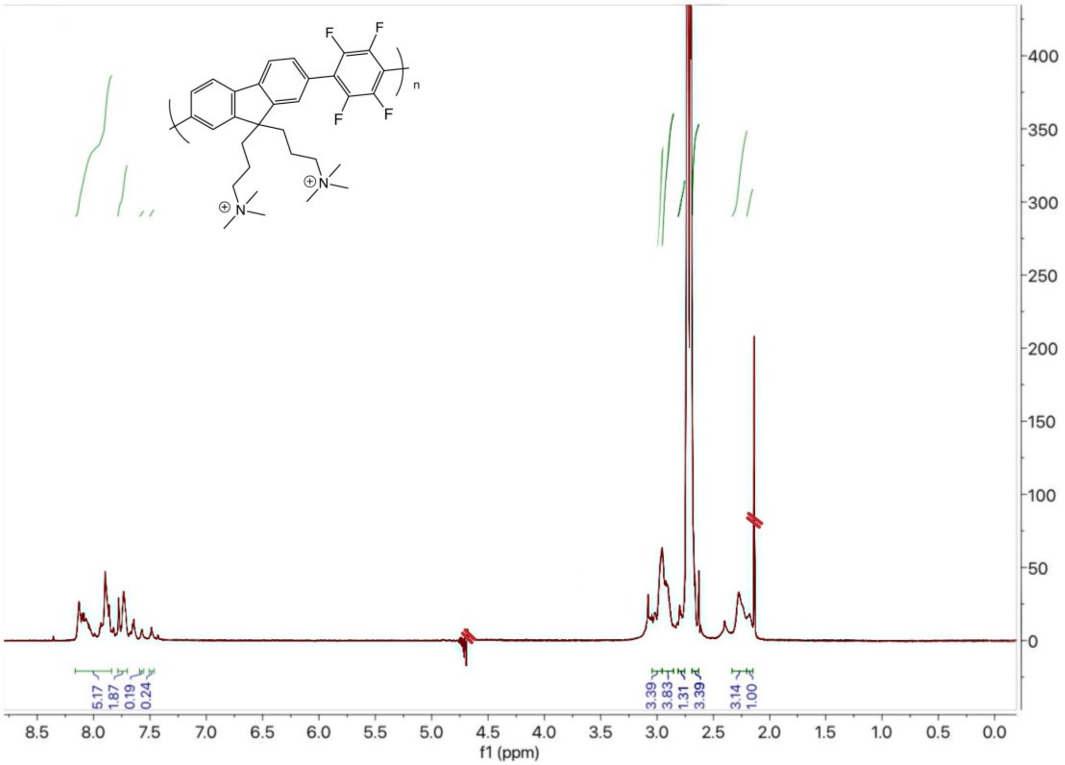

Figure S6: PFNF4 1H 800 MHz NMR in D_2O with a small water suppression peak ~4.7ppm and trace amounts of acetone.

1.11 Synthesis of Neutral Polymer nPFNF4

Polymerization of *3,3'-(2-methyl-7-(5-methylthieno[3,2- b]thiophen-2-yl)-9H-fluorene-9,9-diyl)bis(N,N-dimethylpropan-1-amine).* To a clean pressured vial, 1,4-dibromo-2,3,5,6-tetrafluorobenzene (300 mg, 1.05 mmol), FNB (500 mg, 0.84 mmol), potassium carbonate

(K_2CO_3, 1.25 g, 9.6 mmol), 1,4- dioxane (*Dioxane*, 8 mL, 90.4 mmol), DI H_2O (7.0 mL, 277.0 mmol), and Pd(dppf)Cl_2 (4 mg, 0.005 mmol) were added. The entire reaction vessel, including the solution was flushed with N_2(g). The pressured vial was placed into a silicon oil bath, stirred, and heated at 100 °C for 48 hours. To stop the reaction, the stirring function was turned off and the polymer in the dioxane layer was pipetted into DI H_2O to induce precipitation of nPFNF4, decanted and vacuum filtrated to dry.

1.10 Synthesis Conjugated Polyelectrolyte PFNF4

Conjugated polyelectrolyte *3-(2-methyl-7-(5- methylthieno[3,2-b]thiophen-2-yl)-9-(3-(trimethylammonio)propyl)-9H-fluoren-9-yl)propyl)-l4- azaneyl)methylium*. In-situ quaternization of nPFNF4 was done with the addition of iodomethane (MeI, 4 mL, 62 mmol) directly to the remaining dioxane layer containing nPFNF4 and was left to react for 2 hours before the addition of DI H_2O, to assist in the dissolution of the polymer. The reaction was then heated to 50 °C for 5 days and aliquots of DI H_2O were systematically added until there was no more headspace in the 250mL pressured vial and the polymer was well dissolved. The solution was then dialyzed via the Thermo Scientific™ Slide-A-Lyzer Dialysis Cassette (10,000 MWCO) submerged in a vat of DI H_2O. The DI H_2O was exchanged with fresh DI H_2O every day for 3 days. After 3 days, the dialyzed solution of PFNF4 was concentrated under reduced pressure, filtered, transferred into multiple 25 mL Falcon tubes, and lyophilized to yield PFNF4 as a grey solid (74.6% yield). 1H NMR: δ 2.07-2.20, 2.13, 2.16, 2.30-2.41, 2.35, 2.85-2.94, 3.01-3.13, 3.07, 7.48, 7.49-7.67, 7.72, 7.59, 7.61, 7.73-7.85, 7.79, 8.02-8.14, 8.13.

S2. Neutral Polymer Molecular Weight Calculations

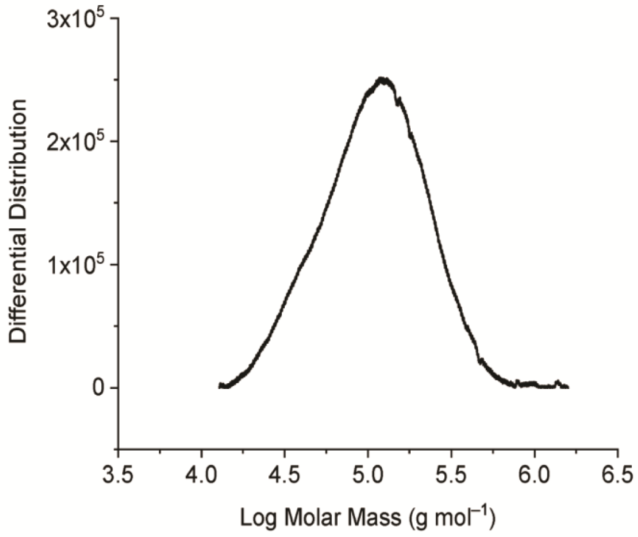

Figure S7. nPFNF2 (a) distribution plot for extraction of neutral conjugated polymer molecular weight, shown in Table S1.

Table S1. Polymer Molecular Weight

Sample	M_n (g/mol)	M_w (g/mol)	M_w/M_n	DP
nPFNF2	84133	139535	1.69	311.05

S3. Obtaining the Energy Transfer Efficiency from the PLE Spectra

To extract relative energy transfer efficiencies from PTAK PLE spectra, we first fit the OD spectrum of a dilute isolated PTAK solution to a sum of 4 Gaussian functions with *unconstrained* fitting parameters over the 300 nm to 700 nm range. These fitting parameters were then used as starting inputs for a *constrained* fit of CPEC PLE spectra. First, for each CPEC, we identified the longest wavelength above which the donor EET

contribution to the PTAK PL was approximately zero such that the PLE in this region was due to directly excited PTAK alone. Then we fit the PLE spectrum above this wavelength cutoff to a sum of 4 Gaussian functions where each fitting parameter (amplitude, center, and width) was allowed to vary by ~10-15% of the values found in the PTAK OD fit. An additional common multiplicative scaling factor was applied identically to each Gaussian fitting function. The need to allow each OD fitting parameter to vary by ~10-15% relative to the OD fit arose because the PTAK spectrum underwent a qualitative change upon binding to a cationic donor CPE. Although a fully constrained fit was also attempted, it was clear that such a functional form was qualitatively inappropriate given the change in PLE peak position and spectrum shape on the red side compared to the isolated PTAK OD spectrum. Fits to the red side of the PLE are shown below as dashed red curves. Above the lower wavelength cutoff the PLE fit is clearly of excellent quality.

We proceeded to subtract the red-side PLE fit from the measured PLE spectrum, obtaining the raw contribution to the PTAK PLE due to EET from the donor CPE (teal dashed curves below). As described in the main text, the integral under the resulting curve is related to the relative EET efficiency. The complication that accompanies this estimation stems from the fact that the behavior of the PLE spectrum in the donor EET region is not known. The fitted PLE on the red side, which forms the background intensity on top of which the donor EET contribution sits, is effectively a reasonable guess.

We aimed to obtain a conservative estimate of the donor EET contribution that would at least partially and systematically account for the fact that the fitted background intensity corresponded to an estimate. To do so, we note that the normalized PTAK OD spectrum (dashed light blue curve below) lies well above the fitted PLE spectrum. Thus, we reasoned that the true PLE background intensity on top of which the donor EET contribution sits would lay somewhere between the fitted spectrum and the measured OD spectrum. To obtain a minimally biased estimate of the relative EET efficiency, we subtracted the (peak-normalized) PLE fit from the (peak-normalized) OD spectrum to form a difference curve which, point by point, represented the effective uncertainty in the true background intensity

of the EET contribution. We thus believed that the least biased estimate was to take the background intensity as equal to half the difference curve. That is, the estimated background intensity at every wavelength was taken to lie exactly halfway between the fitted PLE spectrum and the measured OD spectrum. This contribution was further subtracted from the extracted donor EET peak (teal curve), and the remaining curve was integrated to form E^{rel} as described in the main text.

S4. Photoluminescence Excitation Fit for PFNX:CPECs

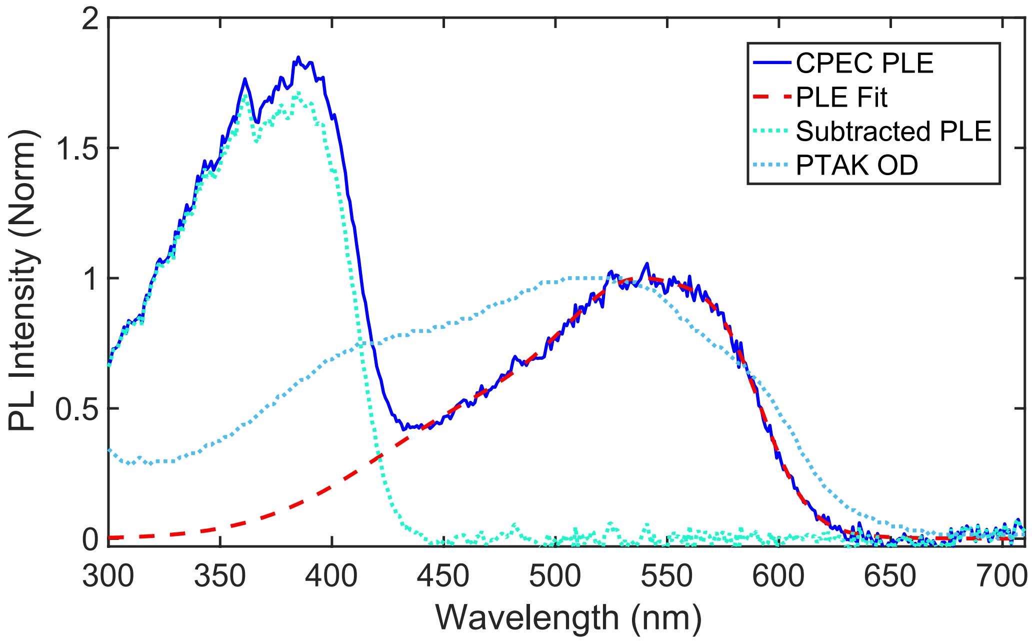

Figure S8. CPEC PLE Fit for PFNB:PTAK and native PTAK OD.

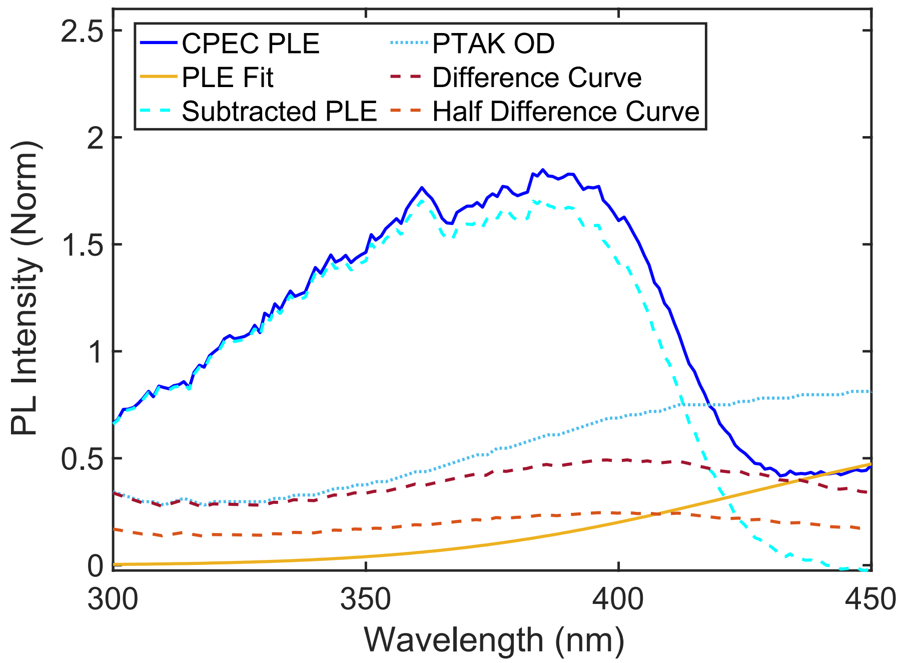

Figure S9. Shown as the blue dashed curve is the calculated PFNB donor contribution to the PLE curve. The red dashed curve is the difference of the peak-normalized experimental PTAK OD and the fitted peak normalized PTAK contribution. The half of this difference curve was used (orange dashed curve) to calculate the background and the error in E_{rel}.

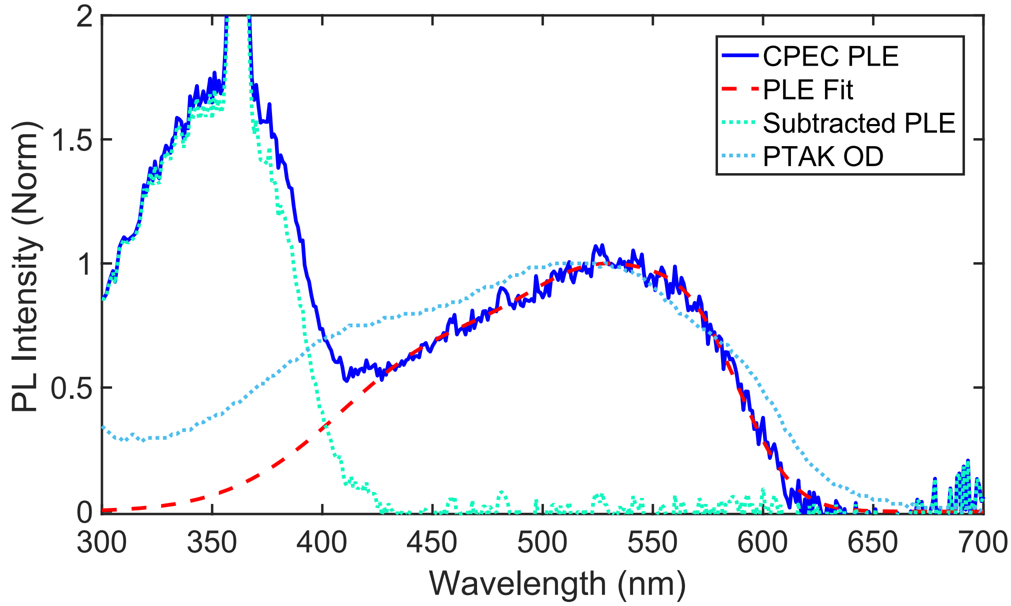

Figure S10. CPEC PLE Fit for PFNF2:PTAK and native PTAK OD.

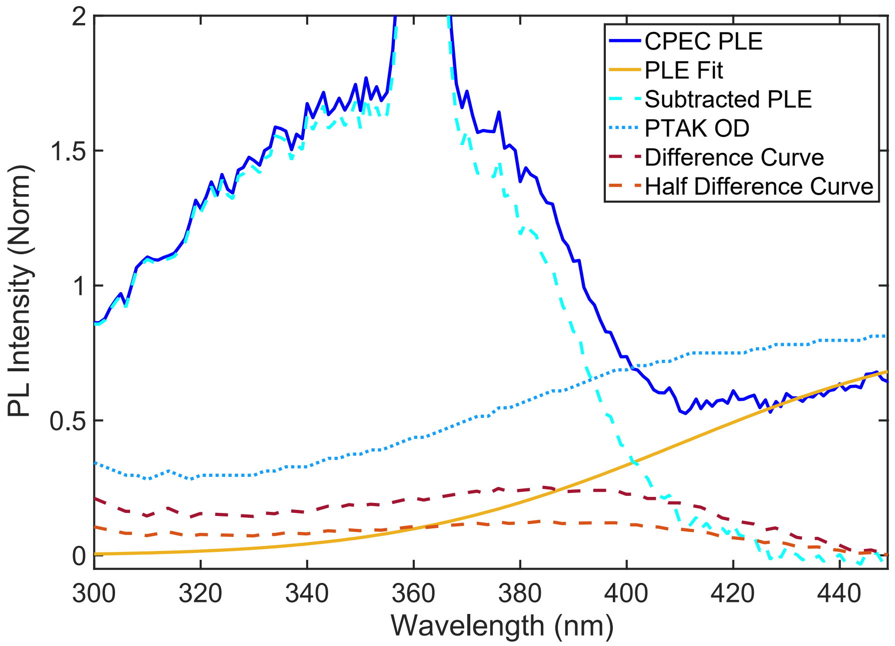

Figure S11. Shown as the blue dashed curve is the calculated PFNF2 donor contribution to the PLE curve. The red dashed curve is the difference of the peak-normalized experimental PTAK OD and the fitted peak-normalized PTAK contribution. The half of this difference curve w was used (orange dashed curve) to calculate the background and the error in E^{rel}.

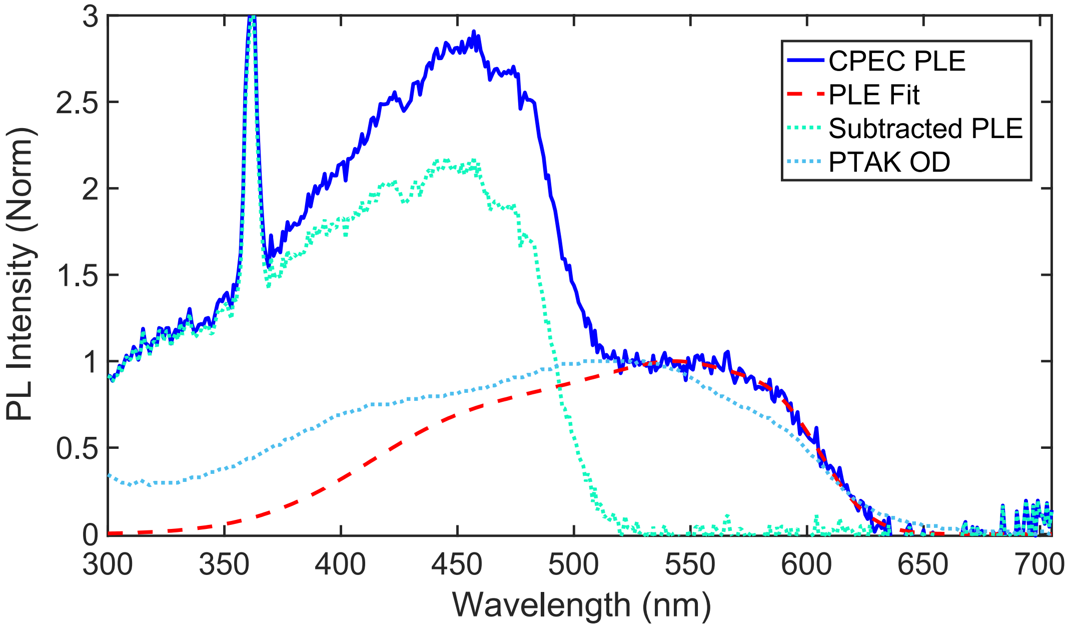

Figure S12. CPEC PLE Fit for PFNT2:PTAK and native PTAK OD.

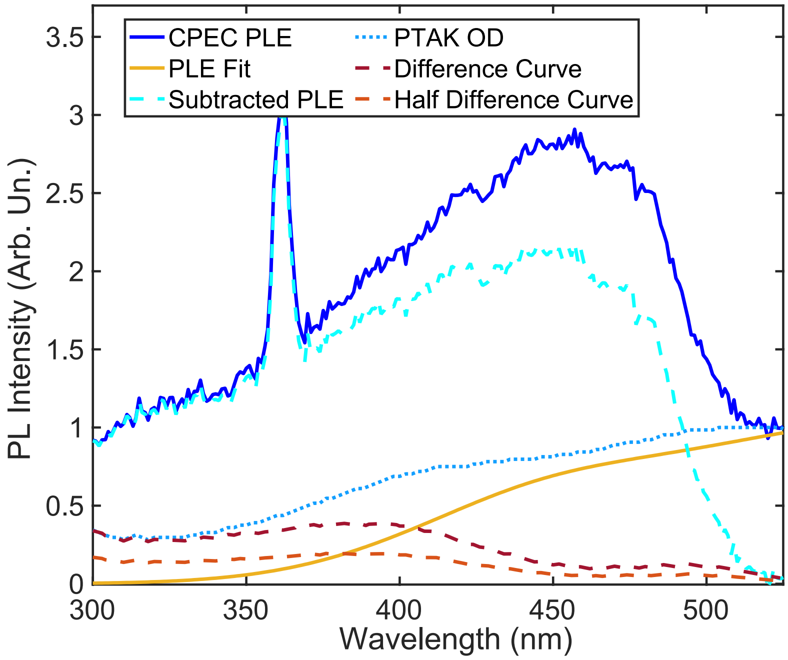

Figure S13. Shown as the blue dashed curve is the calculated PFNT2 donor contribution to the PLE curve. The red dashed curve is the difference of the peak-normalized experimental PTAK OD and the fitted peak-normalized PTAK contribution. The half of this difference curve was used (orange dashed curve) to calculate the background and the error in E_{rel}.

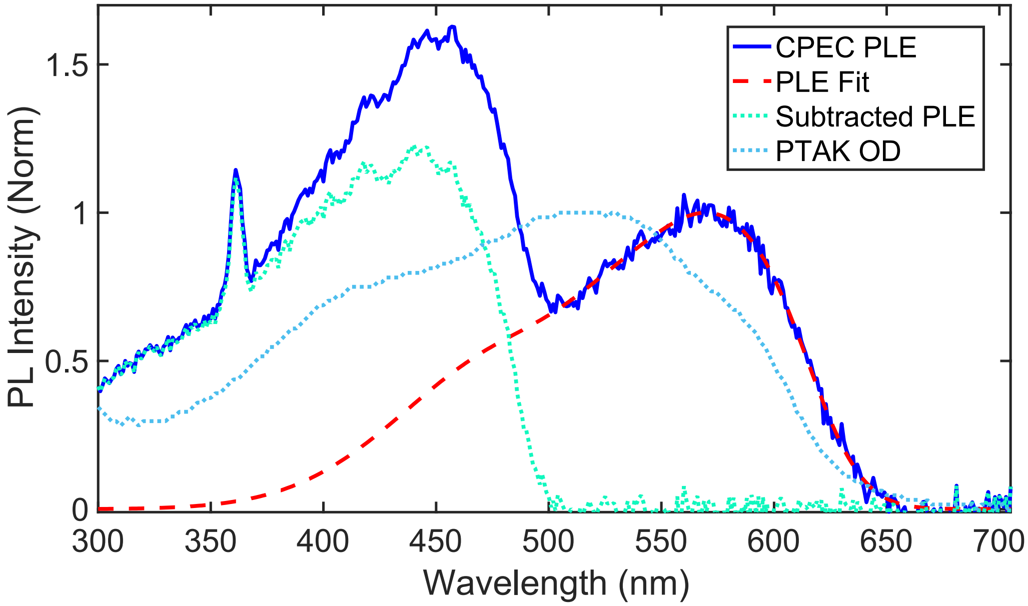

Figure S14. CPEC PLE Fit for PFNT1:PTAK and native PTAK OD.

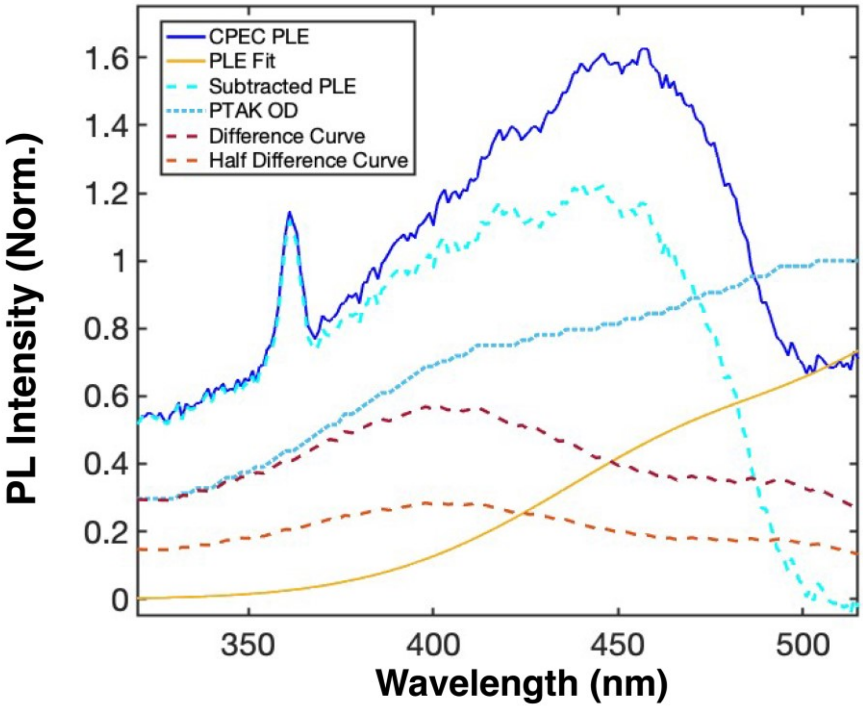

Figure S15. Shown as the blue dashed curve is the calculated PFNT1 donor contribution to the PLE curve. The red dashed curve is the difference of the peak-normalized experimental PTAK OD and the fitted peak-normalized PTAK contribution. The half of this difference curve was used (orange dashed curve) to calculate the background and the error in E^{rel}.

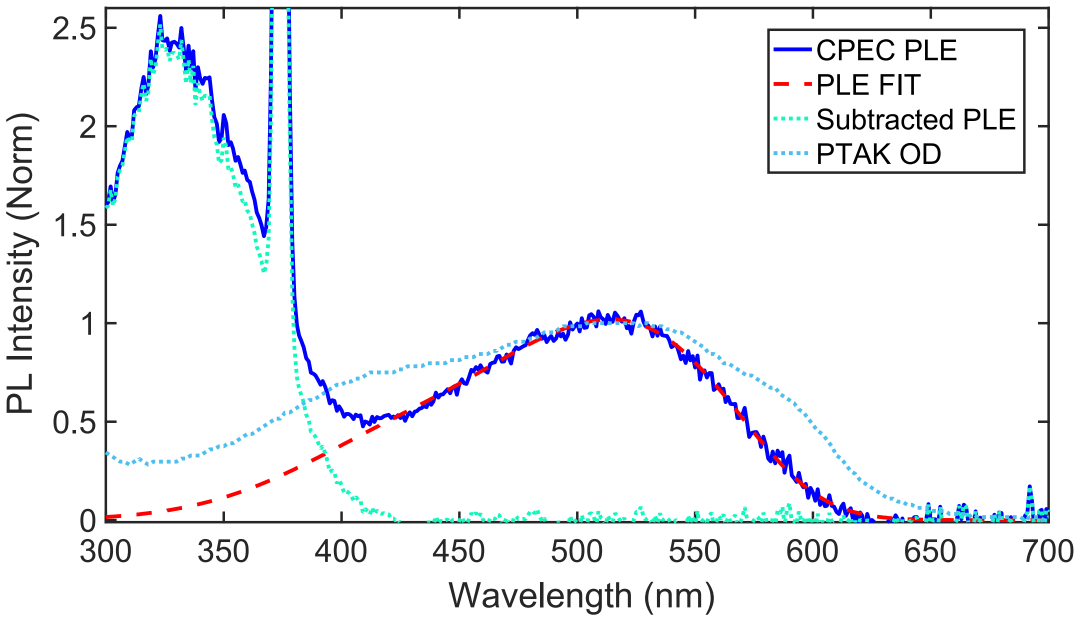

Figure S16. CPEC PLE Fit for PFNF4:PTAK and native PTAK OD.

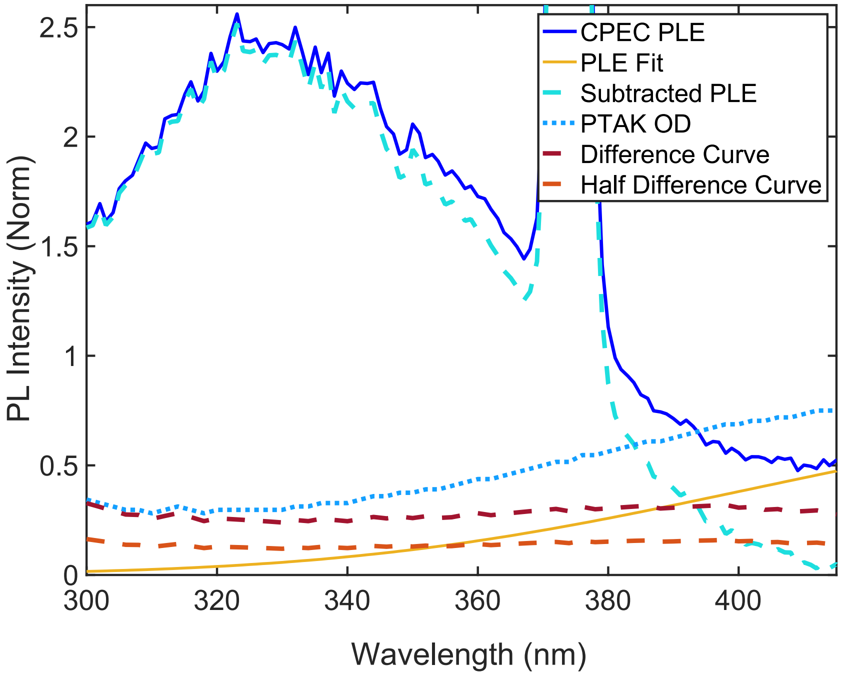

Figure S17. Shown as the blue dashed curve is the calculated PFNF4 donor contribution to the PLE curve. The red dashed curve is the difference of the peak-normalized experimental PTAK OD and the fitted peak-normalized PTAK contribution. The half of this difference curve was used (orange dashed curve) to calculate the background and the error in E^{rel}.

S5. Förster Distance Estimation

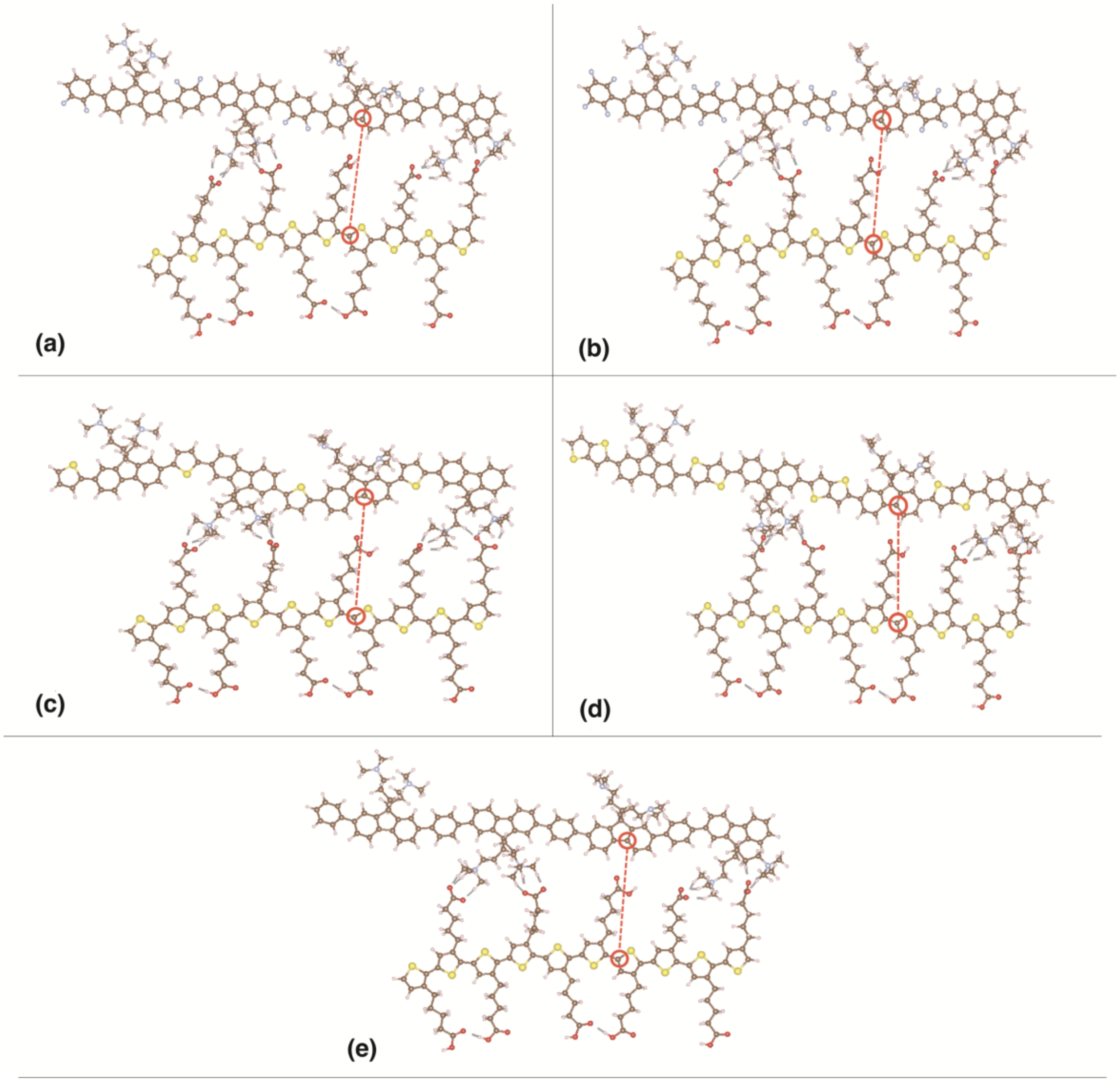

Figure S18. Donor-acceptor CPECs (a) PFNF2 (b) PFNF4 (c) PFNT1 (d) PFNT2, (e) PFNB, optimized by semiempirical method, GFN2-xTB, to extract Forster distance (R).

Table S2. Distance between the two donor and acceptor CPEs as shown in Figure S18.

PFNX-PTAK	Distance (Angstrom)
B	13.2
F2	12.7
F4	13.0
T1	13.7
T2	13.1

S6. Photoluminescence Spectra of PTAK in CPECs

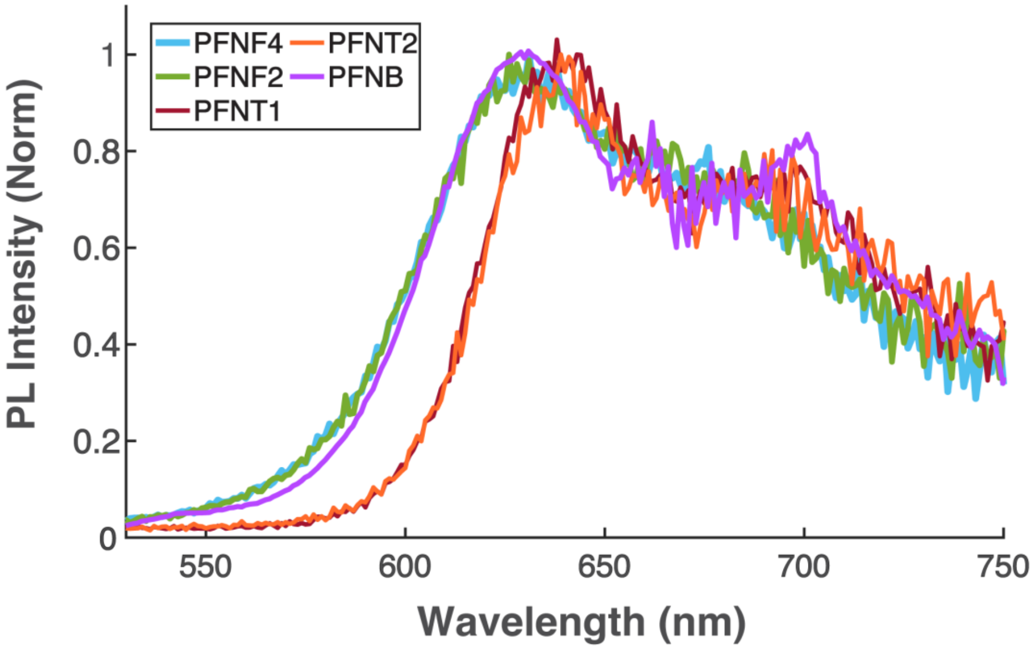

Figure S19. Normalized PL of 70:30 charge ratio of CPECs excited at 545 nm.

S7. Transient Absorption of PFNX:PTAK. CPECs

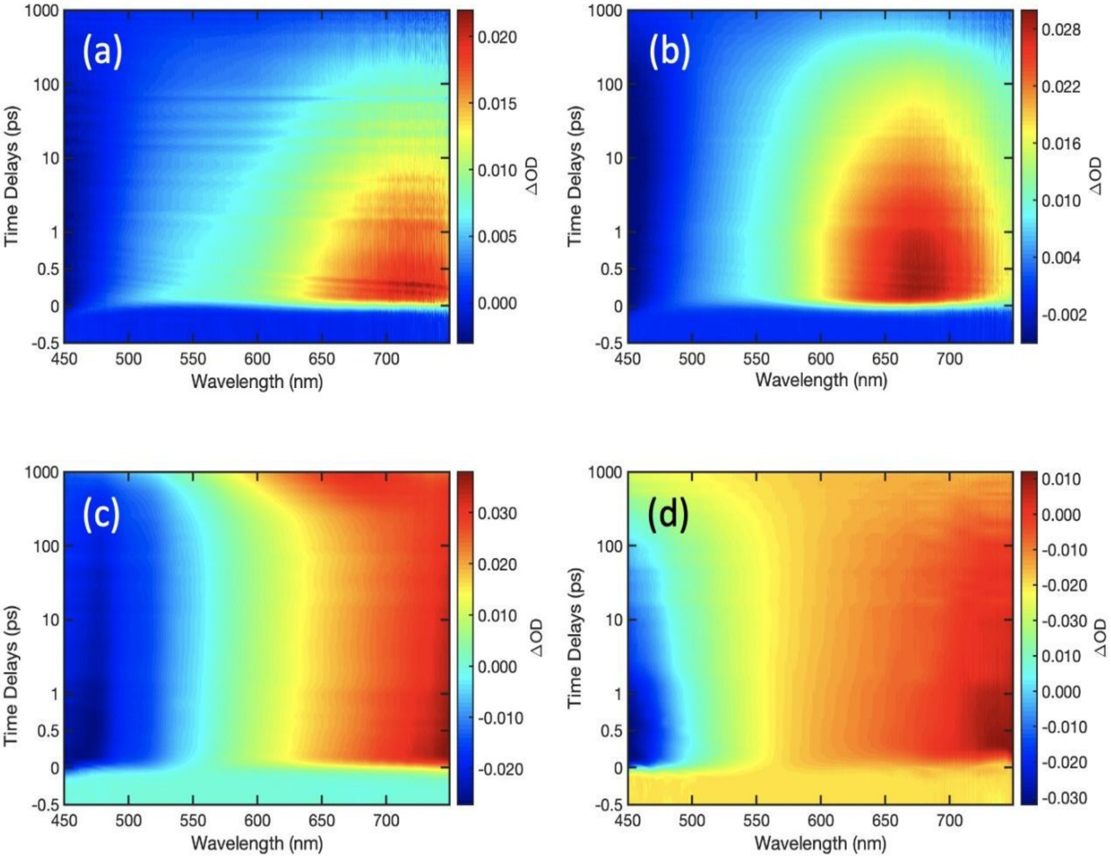

Figure S20. Transient absorption spectra obtained with uncomplexed (isolated) donor CPEs presented in a false-color contour representation: (a) F4, (b) F2, (c) T1, (d) T2. Spectra were collected following excitation near the peak of the steady-state absorption spectrum of each donor (excitation wavelength is indicated in each subpanel). Spectral dynamics are explained in the text. The same data is presented as a spectral waterfall plot in Figure 3 of the main text.

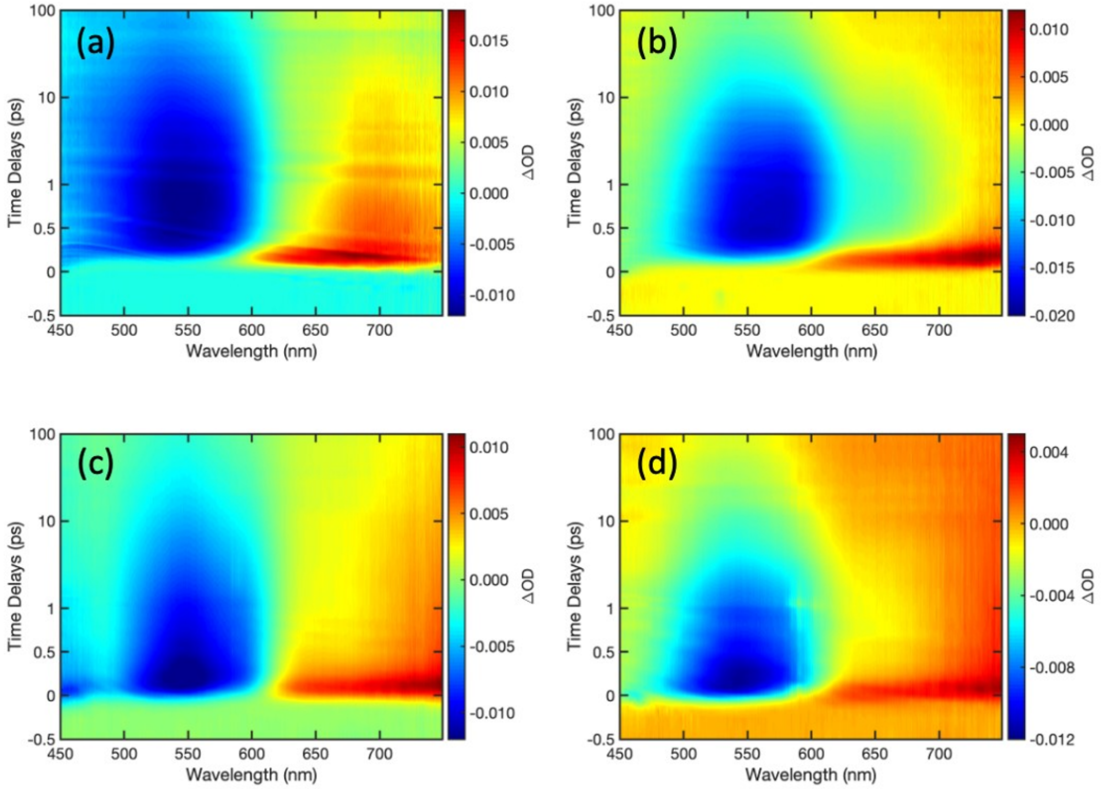

Figure S21. Transient absorption spectra obtained with PFNX:PTAK CPECs presented in a false-color contour representation: (a) F4, (b) F2, (c) T1, (d) T2. Spectra were collected following excitation near the peak of the steady-state absorption spectrum of each donor (excitation wavelength is indicated in each subpanel). Spectral dynamics are explained in the text. The same data is presented as a spectral waterfall plot in Figure 4 of the main text.

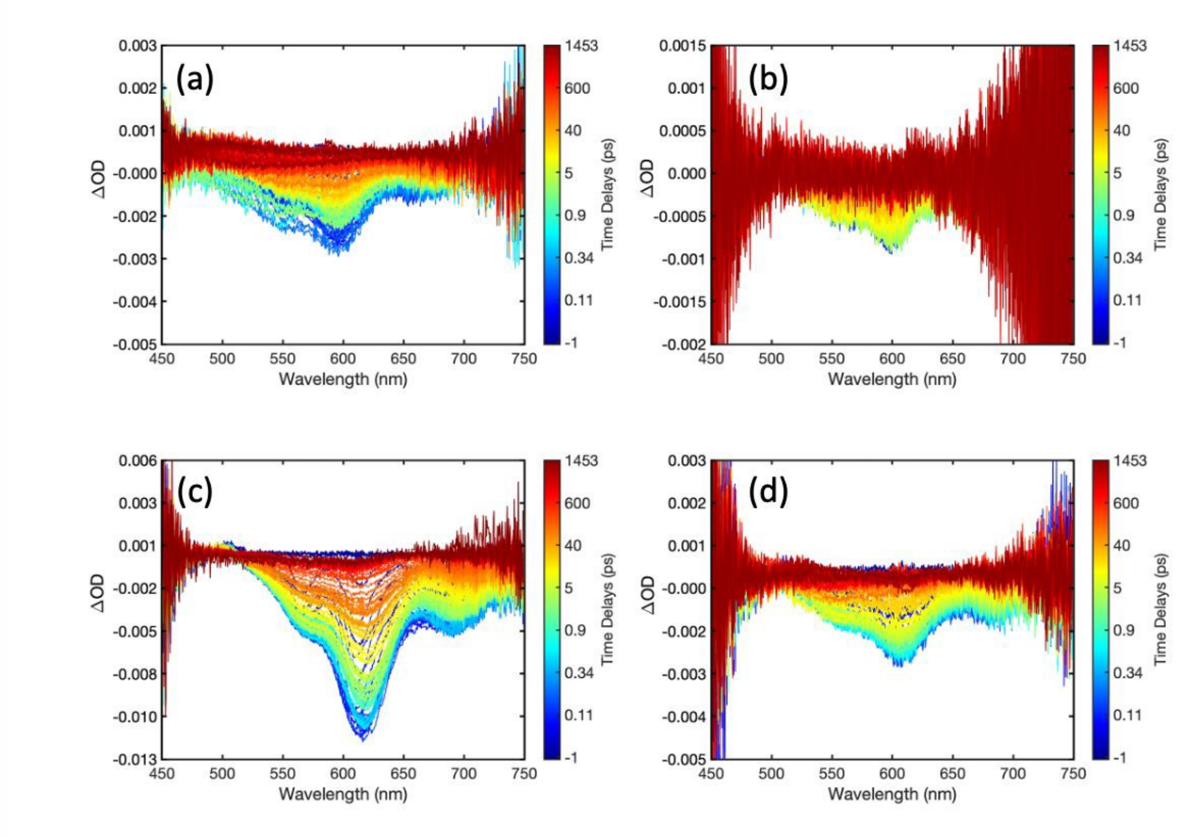

Figure S22. Transient absorption spectra obtained through selective excitation of PTAK in PFNX:PTAK CPECs at 600 nm: (a) F4, (b) F2, (c) T1, (d) T2.

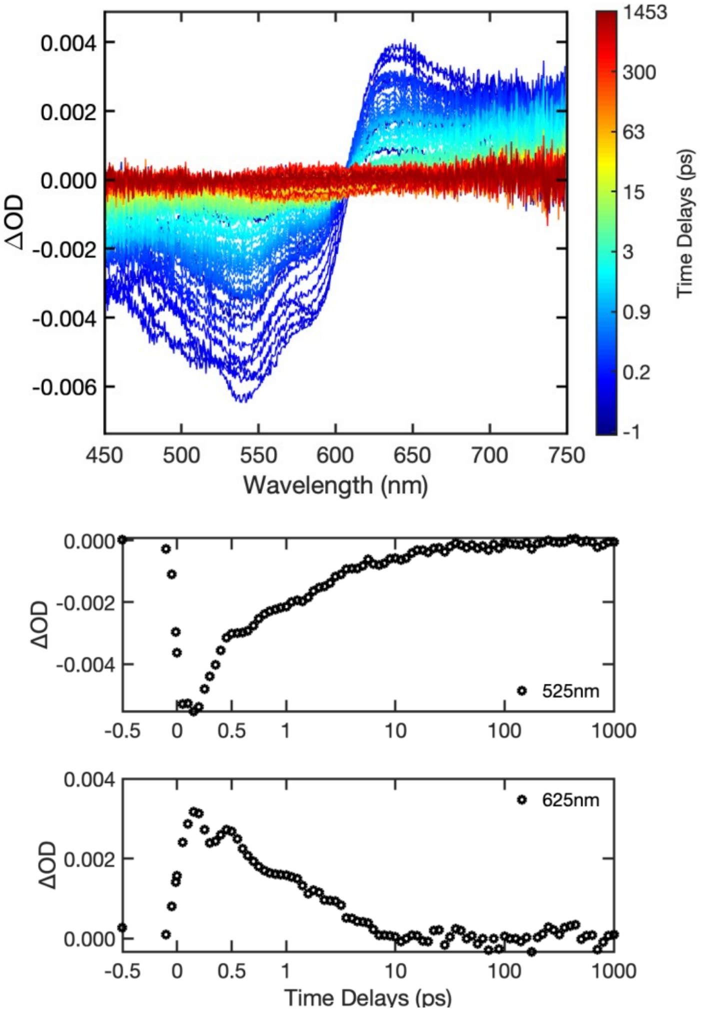

Figure S23. Transient absorption spectra (top) obtained with pure PTAK. Spectra was collected at 425 nm excitation. Time-dependent cuts at selected probe wavelengths (bottom) from TA spectra.

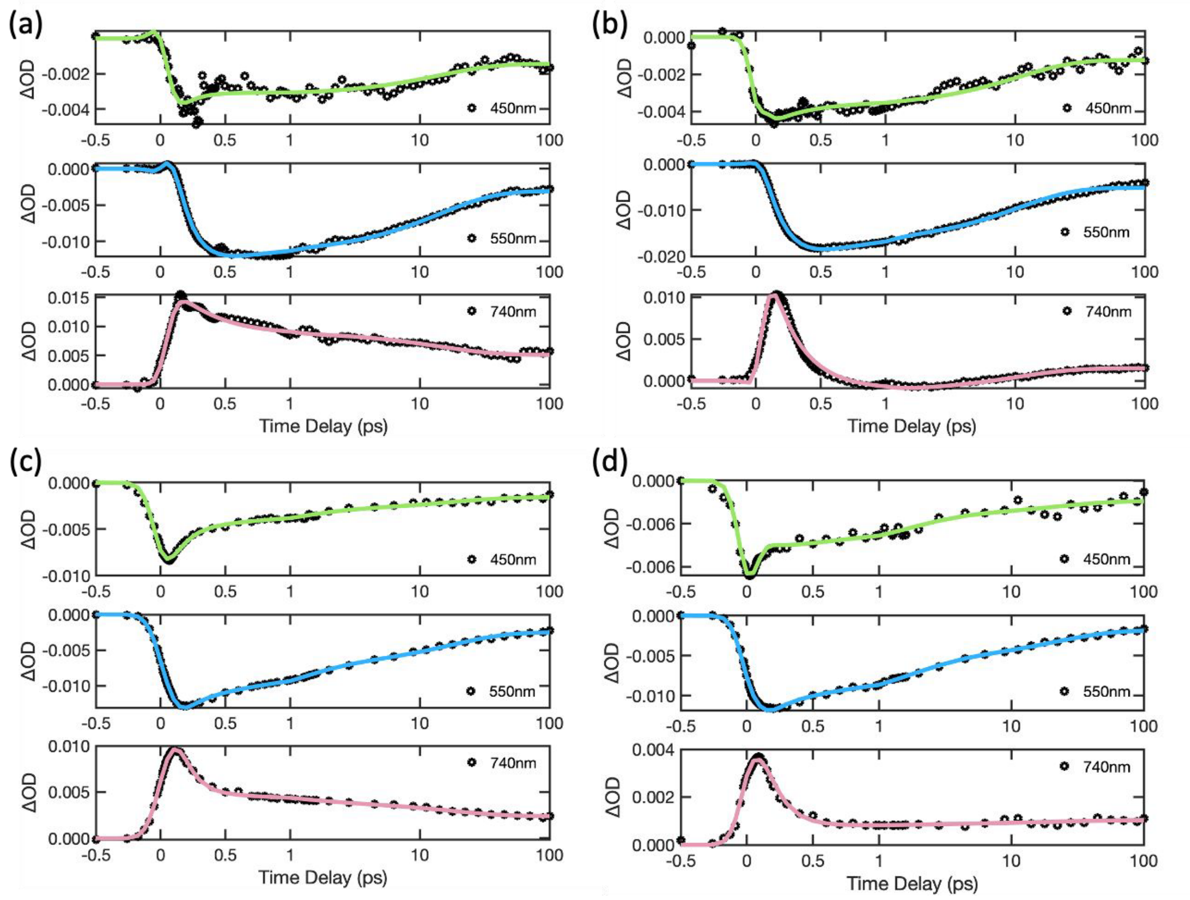

Figure S24. Time-dependence at selected probe wavelengths with excitation of PFNX:PTAK CPECs. (a) F4, (b) F2, (c) T1, (d) T2. Data are shown with symbols, fits determined by global analysis as lines. Lifetimes corresponding with fitted kinetic models are summarized in Table S3.

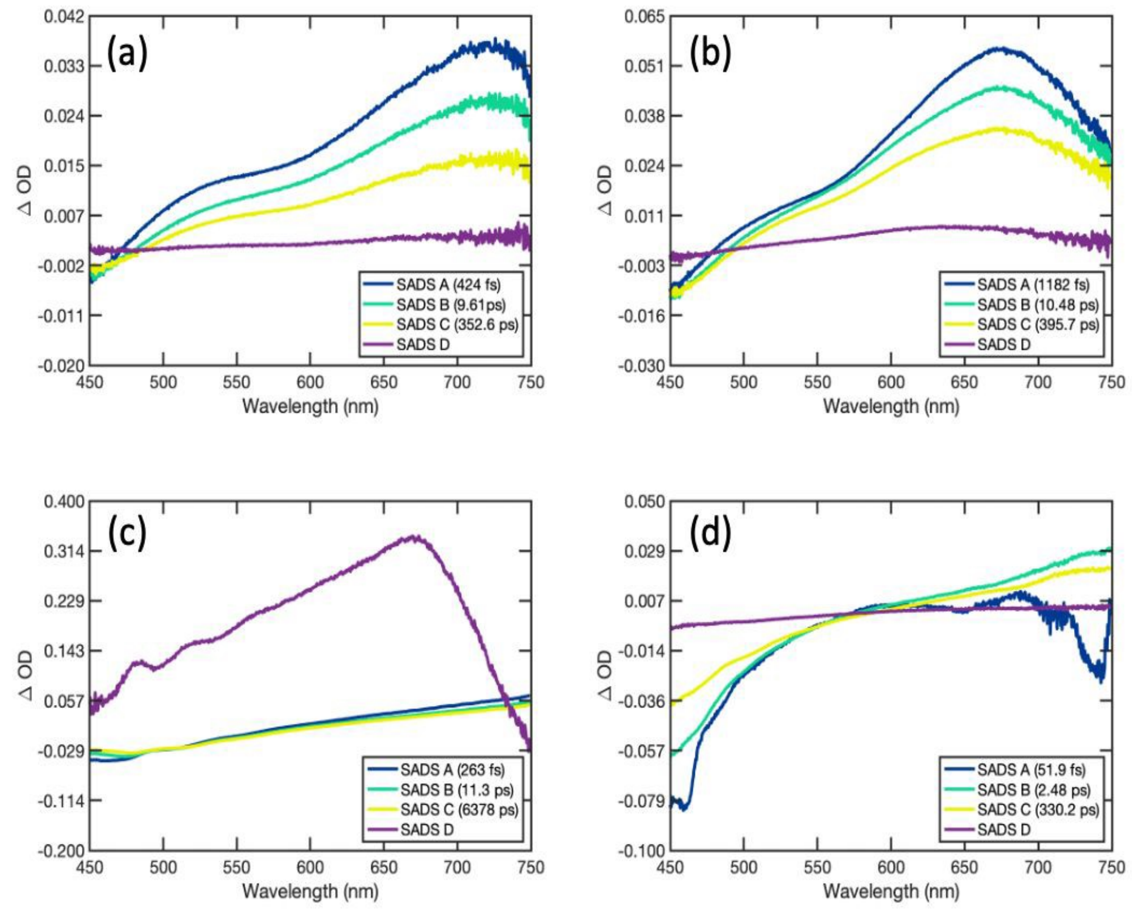

Figure S25. Species Associated Difference Spectra (SADS) obtained from global analysis of transient absorption spectra collected with donor CPEs using the four-state kinetic interconversion model expressed by Equation 5. (a) F4, (b) F2, (c) T1, (d) T2. Lifetimes corresponding with each SADS are listed in Table S1.

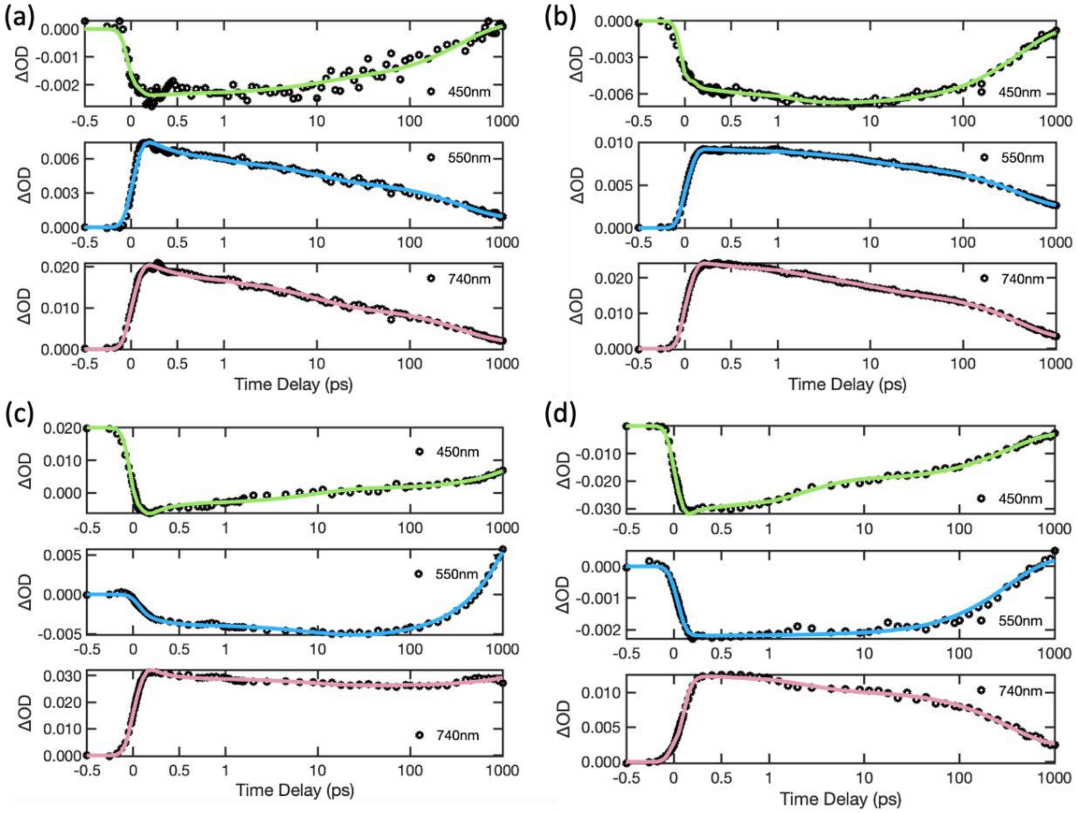

Figure S26. Time-dependence at selected probe wavelengths with excitation of donor CPEs. (a) F4, (b) F2, (c) T1, (d) T2. Data are shown with symbols, fits determined by global analysis as lines. Lifetimes corresponding with fitted kinetic models are summarized in Table S2.

Table S3. Best-fit lifetimes determined from global analysis of TA data collected with PFNX:PTAK CPECs subject to a four-state sequential kinetic interconversion model (Equation 5 of the main text).

	F4	F2	T1	T2
τ_1 **(fs)**	156	236	95.6	134
τ_2 **(ps)**	0.259	0.241	1.0	1.54
τ_3 **(ps)**	14.2	10.6	17.2	21.1

Table S4. Best-fit lifetimes determined from global analysis of TA data collected with donor CPEs subject to a four-state sequential kinetic interconversion model (Equation 5 of the main text).

	F4	F2	T1	T2
τ_1 **(fs)**	424	1180	263	52
τ_2 **(ps)**	9.6	10.5	11.3	2.5
τ_3 **(ps)**	353	396	6380	330

S8. Slow Dynamics and Energy Transfer of PFNX:PTAK Complexes

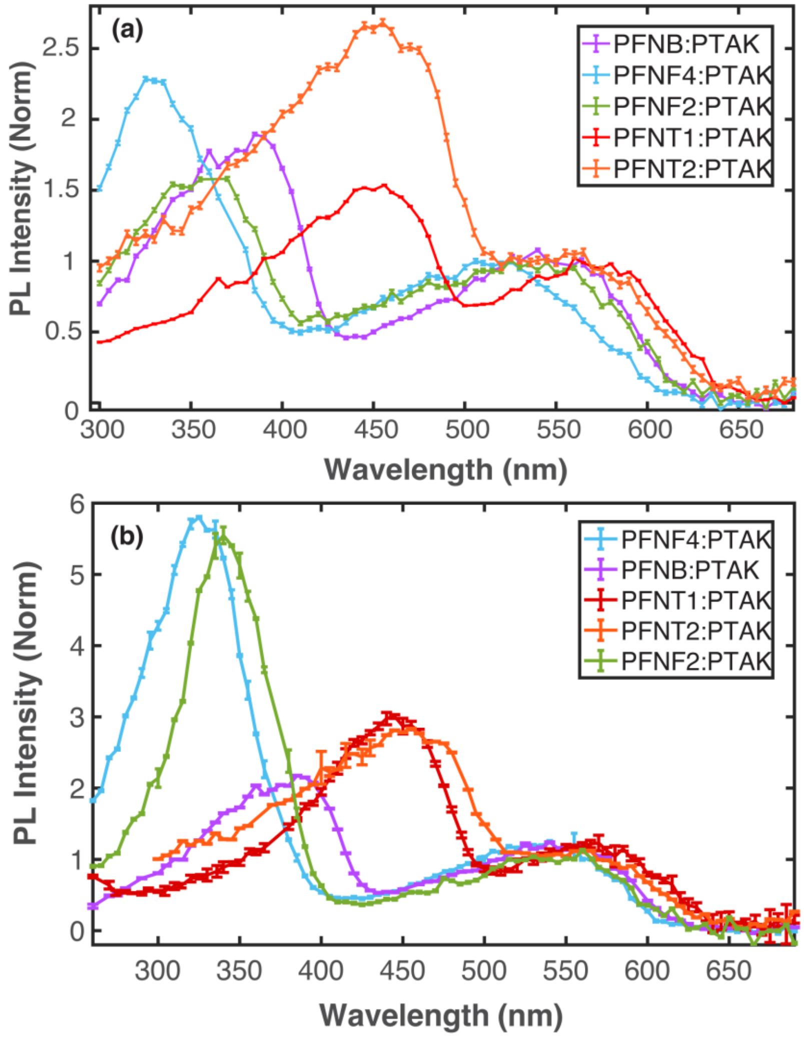

Figure S27. Photoluminescence excitation (PLE) spectra of the PFNX: PTAK series when complexes were (a) freshly made and (b) after 2 weeks.

S9. TRPL Lifetime Parameters

Native CPE	τ_1 (ns)	a_1	τ_2 (ns)	a_2	$\langle\tau\rangle$ (ns)
PFNT1	0.24	0.99	0.9	0.0013	0.24
PFNT2	0.20	0.99	0.67	0.00050	0.20
PFNF2	0.14	0.99	0.43	0.00020	0.14
PFNF4	0.16	0.99	0.51	0.00020	0.16
CPEC	τ_1 (ns)	a_1	τ_2 (ns)	a_2	$\langle\tau\rangle$ (ns)
PFNT1:PTAK	0.12	0.99	1.36	0.0010	0.12
PFNT2:PTAK	0.11	0.99	0.73	0.0003	0.12
PFNF2:PTAK	0.14	0.99	0.91	0.0040	0.14
PFNF4:PTAK	0.15	0.99	0.42	0.0016	0.15

Table S5: Deconvolved PL lifetimes, component amplitudes, and average PL lifetimes.

S10. DFT Calculations

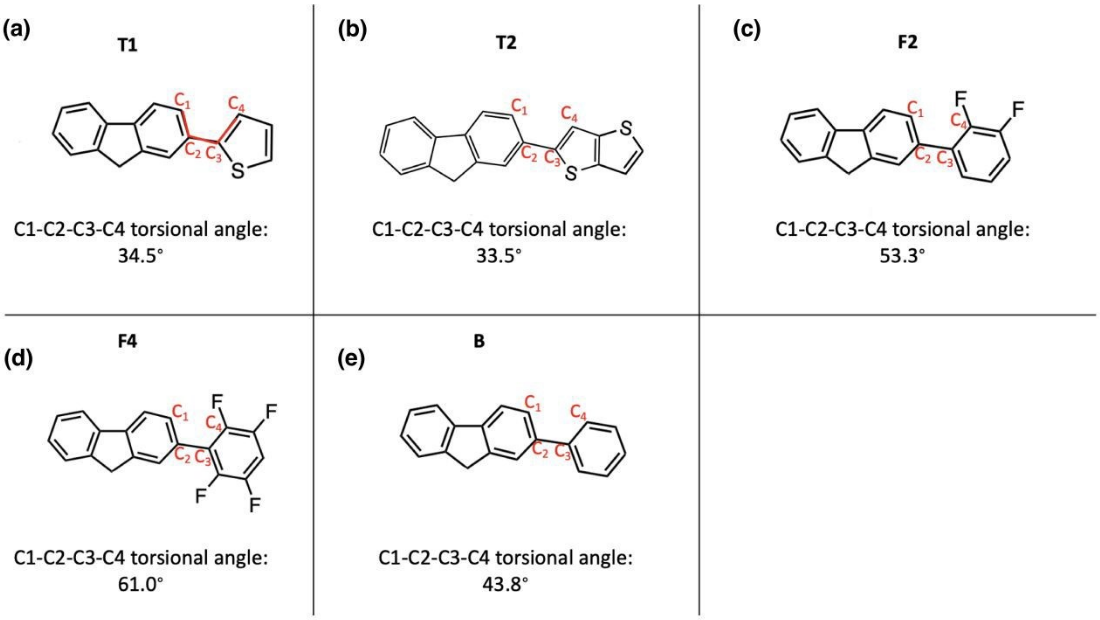

Figure S28. Torsional angles along linkage bonds of the ground-state equilibrium geometries of co-monomer units for PFNT1 (a), PFNT2 (b), PFNF2 (c), PFNF4 (d), and PFNB (e).

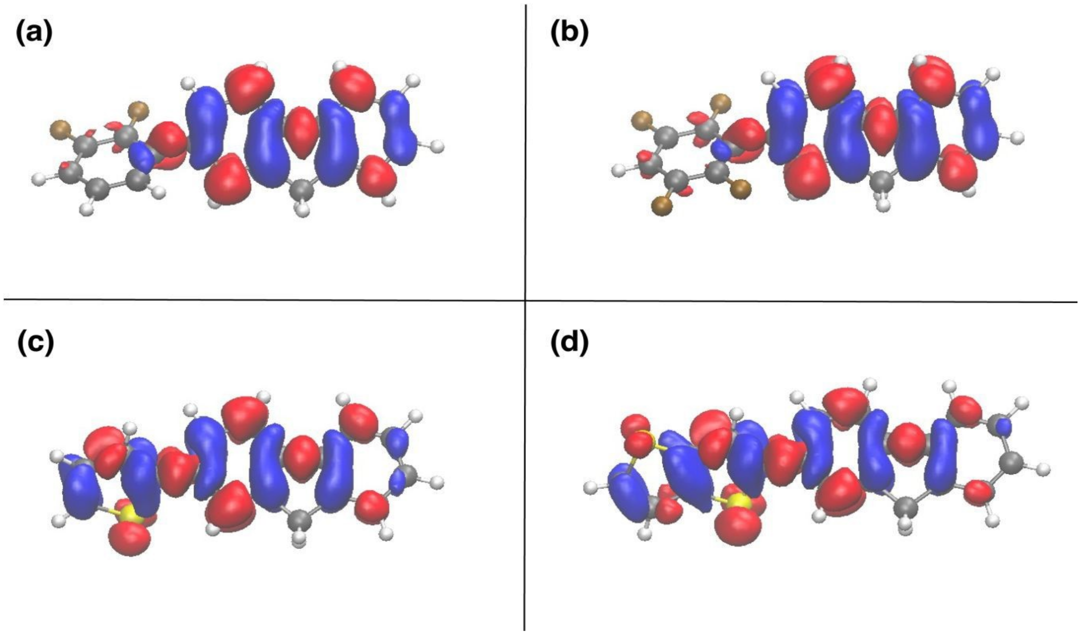

Figure S29. Charge difference density for donor repeat unit of PFNF2 (a), PFNF4 (b), PFNT1 (c),and PFNT2 (d) on the electronic transition from the ground state to the lowest excited state. The blue and red colors correspond to negative and positive isosurfaces, respectively. The isovalue is 8×10^{-4} in a.u.
